# Spatiotemporal localization of jasmonate in the regulation of fruit set in tomato

**DOI:** 10.1093/jxb/eraf349

**Published:** 2025-08-01

**Authors:** Yukako Nomura, Yu Lu, Keiichiro Harada, Hirofumi Enomoto, Ryoichi Yano, Kentaro Ezura, Mikiko Kojima, Yumiko Takebayashi, Hitoshi Sakakibara, Hiroshi Ezura, Tohru Ariizumi

**Affiliations:** Graduate School of Life and Environmental Sciences, University of Tsukuba, Tsukuba, Ibaraki 305-8572, Japan; Faculty of Life and Environmental Sciences, University of Tsukuba, Tsukuba, Ibaraki 305-8572, Japan; Graduate School of Life and Environmental Sciences, University of Tsukuba, Tsukuba, Ibaraki 305-8572, Japan; Department of Biosciences, Teikyo University, Utsunomiya, Tochigi 320-8551, Japan; Advanced Instrumental Analysis Center, Teikyo University, Utsunomiya, Tochigi 320-8551, Japan; Advanced Analysis Center, National Agriculture and Food Research Organization, Tsukuba, Ibaraki 305-8518, Japan; Faculty of Life and Environmental Sciences, University of Tsukuba, Tsukuba, Ibaraki 305-8572, Japan; RIKEN Center for Sustainable Resource Science, Yokohama, Kanagawa 230-0045, Japan; RIKEN Center for Sustainable Resource Science, Yokohama, Kanagawa 230-0045, Japan; Graduate School of Bioagricultural Sciences, Nagoya University, Nagoya, Aichi 464-8601, Japan; Faculty of Life and Environmental Sciences, University of Tsukuba, Tsukuba, Ibaraki 305-8572, Japan; Tsukuba Plant Innovation Research Center, University of Tsukuba, Tsukuba, Ibaraki 305-8572, Japan; Faculty of Life and Environmental Sciences, University of Tsukuba, Tsukuba, Ibaraki 305-8572, Japan; Tsukuba Plant Innovation Research Center, University of Tsukuba, Tsukuba, Ibaraki 305-8572, Japan; University of Sydney, Australia

**Keywords:** Filament, fruit set, gibberellin, jasmonate, mass spectrometry imaging, ovule, parthenocarpy, seedless, *Solanum lycopersicum*, tomato

## Abstract

Tomato (*Solanum lycopersicum*) is a globally important crop, typically requiring pollination for fruit set. Seedless fruit production—fruit set without pollination (parthenocarpy)—is a desirable trait for horticulture, but its molecular mechanisms are not fully understood. Research on tomato fruit set has largely focused on phytohormones such as auxin and gibberellins (GAs), while the role of jasmonic acid (JA) remains unclear. Here, we identified a novel seedless mutant, *Sldad1* (*Solanum lycopersicum defective in anther dehiscence1*) with a mutation in JA biosynthesis induced by ethyl methanesulfonate mutagenesis in the ‘Micro-Tom’ background. The wild type *SlDAD1* was found to be specifically transcribed in the stamen filament, particularly 2 d before anthesis, with JA levels correlating to its transcript abundance, as visualized by imaging mass spectrometry. Notably, JA also accumulated in the ovary and ovule before anthesis. The *Sldad1* mutant produced seedless fruit without any manual pollination or emasculation and seeded fruit via self-pollination. The mutant showed increased cell expansion, elevated GA levels, and higher transcripts of *SlGA20ox3*, while treatment to inhibit GA biosynthesis suppressed *Sldad1*-induced fruit set. Overall, our findings suggest that JA synthesized in the filament accumulates in the ovule before anthesis, thereby preventing tomato fruit set, at least in part, through the modulation of GA metabolism.

## Teaser

The tomato *dad1* mutant, defective in jasmonate biosynthesis, produces seedless fruits under normal conditions, and analysis of its expression shows that spatiotemporal localization of jasmonate regulates fruit set primarily by modulating gibberellin metabolism.

## Introduction

Tomato (*Solanum lycopersicum*) is among the most important crops globally (León-García *et al.*, 2017) and is a model plant for studying fleshy fruit development. Tomato production largely depends on the efficiency of fruit set, the process by which the ovary transforms into fruit. Pollination is normally required for fruit set, but this process can be negatively affected by adverse conditions during the reproductive phase. The formation of seedless fruits, such as those resulting from parthenocarpy without pollination, is a desirable trait in horticultural crops, but its precise molecular mechanisms are still not fully understood.

Tomato fruit development has three primary phases (Gillaspy *et al.*, 1993). In the first phase, cell divisions occur shortly (1–2 d) before anthesis, after which an ‘ovary arrest’ state occurs. The second phase marks the establishment of fertilization and the onset of active cell division, a critical period that determines whether the fruit will proceed to cell division and enlargement. The success of fruiting depends on the initiation of this phase. In the third phase, cell enlargement surpasses cell division, allowing the fruit to reach its final size before ripening begins, and it eventually turns red. While the transition to the second phase, which includes pollination and fertilization, is normally required, this mechanism appears to be by-passed in seedless fruits (parthenocarpy), although the underlying mechanisms remain unclear.

Various phytohormones play roles in tomato fruit set (Serrani *et al.*, 2007; Matsuo *et al.*, 2012; Ariizumi *et al.*, 2013; Ding *et al.*, 2013; Shinozaki *et al.*, 2015; Ezura *et al.*, 2023). The regulation of fruit development is primarily influenced by auxins and gibberellins (GAs), with GAs thought to function as the downstream regulator of fruit set in tomato. Previous studies have suggested roles for additional phytohormones, including cytokinins, abscisic acid, and ethylene, in tomato fruit set (Shinozaki *et al.*, 2015). Several seedless mutants have been generated through ethyl methanesulfonate (EMS) mutagenesis within the genetic background of the miniature cultivar ‘Micro-Tom’ (Saito *et al.*, 2011). The parthenocarpic mutant *Slarf8* suggests crosstalk between jasmonic acid (JA) and auxin signaling (Israeli *et al.*, 2023), whilst the *Slagl6* and *Slhb15a* mutants exhibit ovule defects and show parthenocarpy under adverse temperature conditions (Klap *et al.*, 2017; Clepet *et al.*, 2021; Gupta *et al.*, 2021).

In addition to auxin and GA, JA has been implicated in fruit initiation. It plays a critical role in regulating plant defense responses, growth, and development (Browse, 2009a), and is activated in response to pathogens, insect attacks, and wounding stress. Additionally, JA is an important regulator of floral development and fertility, including stamen development and sex determination (Yuan and Zhang, 2015; Acosta and Przybyl, 2019; Browse and Wallis, 2019).

In the JA biosynthesis pathway, the initial step is thought to occur *de novo* in the plastids (Ishiguro *et al.*, 2001). α-linolenic acid (α-LA) is produced by fatty acid desaturase (FAD) and released from the membrane by phospholipase A1 (PLA). This compound is subsequently processed by lipoxygenase (LOX), followed by conversion to 12-oxo-phytodienoic acid (OPDA) by allene oxide synthase (AOS) and allene oxide cyclase (AOC). OPDA is then transported from the plastid to the cytoplasm by the channel-like protein JASSY (Guan *et al.*, 2019; Wasternack and Hause, 2019) and partially directed to peroxisomes by the ABC transporter COMATOSE (CTS) (Theodoulou *et al.*, 2005). Within peroxisomes, OPDA undergoes conversion to OPC-8:0 by OPDA reductase 3 (OPR3), followed by addition of CoA via OPC-8:0 CoA ligase (OPCL), and is further processed by acyl-CoA oxidase (ACX), multifunctional protein (MPF), and 3-keto-acyl-CoA thiolase (KAT) (Chini *et al.*, 2018; Wasternack and Hause, 2018). The bioactive form, (+)-7-iso-jasmonoyl-L-isoleucine (JA-Ile), is recognized by the JA co-receptor complex comprising the F-box protein CORONATINE INSENSITIVE1 (COI1) and the JASMONATE ZIM DOMAIN (JAZ) protein.

In *Arabidopsis*, mutants deficient in JA biosynthesis, such as *defective in anther dehiscence1* (*Atdad1*), *allene oxide synthase*/*delayed-dehiscence2* (*Ataos*/*dde2*), *allene oxide cyclase* (*Ataoc*), *12-oxophytodienoic acid reductase 3*/*delayed dehiscence1* (*Atopr3*/*dde1*), or JA-insensitive mutants such as *coi1* exhibit male sterility due to impaired stamen filament elongation, delayed anther dehiscence, and reduced pollen viability, which can be restored by methyl jasmonate (Me-JA) treatment (Sanders *et al.*, 2000; Stintzi and Browse, 2000; Ishiguro *et al.*, 2001; von Malek *et al.*, 2002; Browse, 2009b). In rice (*Oryza sativa*), *EXTRA GLUME1* (*EG1*), which is homologous to *AtDAD1*, confers male sterility in the *eg1-1* mutant (Li *et al.*, 2009; Cai *et al.*, 2014). Similarly, mutations in *OsAOC* (the *coleoptile photomorphogenesis 2*/*hebiba* mutant; Riemann *et al.*, 2013) and disruptions in *OsOPR7* lead to male sterility, which is restored by exogenous Me-JA treatment (Tani *et al.*, 2008; Pak *et al.*, 2021). Male sterility also occurs in the rice *jasmonate resistant1* mutant (Riemann *et al.*, 2008; Xiao *et al.*, 2014). In *Cucurbita pepo*, the JA-deficient mutant *lox3a* exhibits parthenocarpy (Cebrián *et al.*, 2023).

In tomato, JA influences female fertility and flower development. Mutations or gene-silencing of JA biosynthesis components such as *SUPPRESSOR OF PROSYSTEMIN RESPONSE2* (*SlSPR2*)/*SlFAD7* (Li *et al.*, 2003, 2004), *SlAOC* (Goetz *et al.*, 2012), and *SlOPR3* (Scalschi *et al.*, 2015) result in female sterility due to impaired female development. The *JA-insensitive 1-1* mutant (*jai1-1*), which affects JA perception by the tomato homolog of Arabidopsis COI1, also results in female sterility, triggering parthenocarpy (Li *et al.*, 2004). Previous research suggests that the R2R3-MYB transcription factor SlMYB21, which mediates a positive feedback loop in JA biosynthesis, enhances JA biosynthesis in tomato carpels and ovules through auxin and GA biosynthesis and signaling (Schubert *et al.*, 2019). The tomato *jai1-1* and *Slmyb21* mutants show similar phenotypes, such as delayed flowering (Feys *et al.*, 1994; Stintzi and Browse, 2000; Caldelari *et al.*, 2011; Niwa *et al.*, 2018). In Arabidopsis, *AtMYB21* provides negative feedback to JA biosynthesis (Reeves *et al.*, 2012; Huang *et al.*, 2017) and is involved in the elongation of stamen filaments and petals (Mandaokar *et al.*, 2006; Song *et al.*, 2011; Reeves *et al.*, 2012). Knockout mutants of *SlMYB21* exhibit reduced JA levels and result in parthenocarpy (Schubert *et al.*, 2019). However, the detailed molecular mechanism underlying tomato fruit set by JA is poorly understood. In this study, we identified and characterized a novel seedless tomato mutant, *Sldad1*, deficient in JA biosynthesis and demonstrated the role of the JA biosynthesis gene *SlDAD1*. We propose a molecular model of early tomato fruit development mediated by JA.

## Materials and methods

### Plant materials and growth conditions

This study used the tomato (*Solanum lycopersicum*) wild type (WT) cultivar ‘Micro-Tom’, which is a dwarf and rapid-growth variety, and *Sldad1* (*W2939*) lines produced from ‘Micro-Tom’ subjected to EMS mutagenesis (Saito *et al.*, 2011). The JA-deficient *tap3* mutant background has been described previously (Okabe *et al.*, 2019). F_2_ mapping populations were derived by crossing *Sldad1* with the *S. lycopersicum* cultivar ‘Resina’. Seeds for ‘Micro-Tom’ (TOMJPF00001), *Sldad1* (TOMJPW2939), ‘Regina’, and ‘Ueleie 106 WP’ were obtained from the National BioResource Project, MEXT, Japan (https://nbrp.jp/en/about-en/).

Seeds were either placed on filter paper wetted with deionized water for 1 week at 25 °C under 16/8 h light/dark, or surface-sterilized with 10% bleach for 5 min and then plated on half-strength Murashige and Skoog (MS) medium (Sigma-Aldrich) enriched with 3% sucrose and incubated at 24  °C under 16/8 h light/dark. For plants in the ‘Micro-Tom’ background and genome-edited plants (see below), germinated seeds were transplanted onto rockwool cubes (50×50×50 mm; Grodan) and cultured in nutrient solution (Ohtsuka house 1 and 2, OAT Agrio Co., Ltd) with an electrical conductivity (EC) of 1.5 dS m^−1^ under conditions of 25 °C with 16/8 h light/dark under fluorescent lights at 200–320 µmol m^−2^ s^−1^. The F_2_ and F_3_ plants were grown in a greenhouse under natural daylight at the University of Tsukuba, Japan, between March 2017 and August 2018.

For fruit phenotyping, the second and third inflorescences were used and fruits were removed to leave only five per plant. Since non-emasculated *Sldad1* flowers appeared to remain unpollinated even after anthesis, while those of WT were self-pollinated, WT flowers were emasculated to prevent pollination in this study. All selected flowers were emasculated at 1 d before anthesis to prevent self-pollination except for those grown in the greenhouse, which were emasculated at 2 d before anthesis. Alternatively, the flowers were manually self-pollinated on the day of anthesis (i.e. 0 days after anthesis, DAA). All samples collected at 0 DAA and earlier were from non-emasculated flowers. Samples were hand-sectioned, photographed, and ovary diameter was measured using the ImageJ software (v.1.52a).

### Identification of the candidate gene

The F_2_ population was mapped by crossing *Sldad1* in the ‘Micro-Tom’ background with cv. ‘Resina’. Twelve seedless plants from the F_2_ population were genotyped using DNA markers across chromosome 10. The whole-genome sequence of the *Sldad1* mutant was obtained using HiSeq X Ten and HiSeq 2000 next-generation sequencing (Illumina). ‘Heinz 1706’ (SL2.5 and SL3.0) and ‘Micro-Tom’ Japan served as reference genomes (Ariizumi *et al.*, 2014; Kobayashi *et al.*, 2014). Linkage analysis was performed using F_2_ plants derived from crosses between the *Sldad1* mutant and the ‘Micro-Tom’ WT or ‘Resina’.

The genome and amino acid sequences were obtained from the Solonaceae Genomics Network (SGN) (https://solgenomics.net/). The SlDAD1 protein domain prediction was performed using Pfam (https://pfam.org/). Orthologs with a total score >200 were selected, and a phylogenetic tree was constructed with multiple-sequence alignment using CLUSTALW (https://www.genome.jp/tools-bin/clustalw/). The sequence information was verified via a BLAST search of the Arabidopsis Information Resource (https://blast.ncbi.nlm.nih.gov/Blast.cgi).

### Plasmid construction and plant transformation

For CRISPR/Cas9 editing, two gRNAs (gRNA1 and gRNA2) were designed using CRISPR-P 2.0 (http://crispr.hzau.edu.cn/CRISPR2/) and CRISPRdirect (https://crispr.dbcls.jp/). The PCR primers used for plasmid construction and determination of transgenic lines are listed in Supplementary Table S1. Each construct was injected into *Agrobacterium* strain GV2260, and ‘Micro-Tom’ plants were transformed using the *Agrobacterium*-mediated leaf disk method as described previously (Sun *et al.*, 2006). The diploid plants were selected using ploidy analysis, and the transgenes were detected by evaluating *NEOMYCIN PHOSPHOTRANSFERASE II* (*NPTII*).

For subcellular localization, the coding sequence (CDS) of *SlDAD1* was cloned into the pGW5 vector and transiently introduced into *Nicotiana benthamiana* leaves. The p19 empty vector was used as a control. Fluorescence of the green fluorescence protein (GFP) was visualized using a confocal laser-scanning microscope (LSM700; Zeiss), and combined images were obtained using the Zeiss ZEN 2009 software.

### Histological analysis

Ovary sections (10 µm) were obtained and observed according to the protocol of Chusreeaeom *et al.* (2014). All measurements were performed on three independent plants, with three sections per ovary/fruit for each plant using the Olympus cellSens imaging software v.1.6. The number of cell layers was determined and the cell size was measured as described previously (Serrani *et al.*, 2007). Three sections were evaluated for each ovary at the same stage.

### Pollen germination assays

Pollen germination assays were performed using liquid germination medium according to the protocol of Kuwabara *et al.* (2022), with slight modifications. Anthers at 0 DAA were sampled (two per 1.5 ml tube) and soaked in 500 μl of liquid germination medium [15.1% w/v polyethylene glycol (average molecular weight 6000), 10% w/v sucrose, 1.63 mM H_3_BO_4_, 1.27 mM Ca(NO_3_)_2_, 1 mM MgSO_4_, 1 mM KNO_3_, 0.1 mM K_2_HPO_4_, pH 7.0] and strongly vortexed to release the pollen from the anthers. To examine viability, the pollen was incubated in the germination medium using a rotator at 25 °C for 24 h, after which viability was determined according to the presence of pollen tubes.

For *in vivo* pollen germination assays on the stigma, pistils were fixed in ethanol:acetic acid (3:1, v/v) solution 2 d nd 4 d after self-pollination. The fixed pistils were soaked in 5 M NaOH for 24 h. After washing three times with deionized water the pistils were stained with 0.001% w/v Aniline Blue in 0.1 M K_2_HPO_4_ buffer (pH 10) for 24 h in the dark. Pollen tubes were observed using a BX53 microscope and digital images were captured using a DP72 camera (both Olympus).

### RNA isolation and quantitative RT-PCR analysis

Filaments and ovaries were collected, immediately frozen in liquid nitrogen, and stored at −80 °C until use. Total RNA was extracted from the samples using an RNeasy Plant Mini Kit (Qiagen, 74904), and genomic DNA was removed using an RNase-free DNase Set (Qiagen, 79254). cDNA was then generated from 1 μg of the total RNA using a SuperScript III First-Strand Synthesis System (Invitrogen). Quantitative reverse-transcription (qRT)-PCR was performed with CFX96 (Bio-Rad), using SYBR Premix Ex Taq II (Tli Rnase H Plus) (Takara Bio, RR820) with specific primers to amplify the gene-coding region (Supplementary Table S1). For the analysis of samples after emasculation, cDNA was generated from the total RNA using ReverTra Ace qPCR RT Master Mix (Toyobo, FSQ-201), and qRT-PCR was performed using TB Green Premix ExTaq II (Tli Rnase H Plus) (TaKaRa Bio, RR820). The following PCR protocol was applied: 95 °C for 30 s, followed by 40 cycles of 95 °C for 5 s and 60 °C for 30 s. The relative expression levels were normalized to the expression of *SAND* (Expósito-Rodríguez *et al.*, 2008) and calculated using the 2^−ΔΔ*C*T^ method (Pfaffl, 2001). Three or four biological replicates were used in the analysis.

### Measurement of endogenous phytohormones

For each replicate sample of the WT and *Sldad1* mutant, at least 100 mg of fresh filaments at −2, −1, and 0 DAA and fresh ovaries at −2, −1, 0, 2, and 4 DAA, with or without pollination, were collected and immediately frozen in liquid nitrogen. Three biological replicates were used for filament samples and four were used for ovary samples from −2 to 0 DAA. Endogenous phytohormones were measured as previously described (Kojima *et al.*, 2009; Shinozaki *et al.*, 2015) without MS probe modification. JA/JA-Ile, auxin (indole-3-acetic acid, IAA), gibberellins (GAs), and abscisic acid (ABA) were quantified by ultra-high performance LC (UHPLC)–electrospray interface (ESI)–quadrupole orbitrap MS (UHPLC/Q-Exactive™; Thermo Scientific) using an ODS column (ACQUITY Premier HSS T3 Column with VanGuard FIT, 1.8 μm, 2.1×100 mm; Waters) with linear gradients of solvent A (0.06% acetic acid) and B (0.01% formic acid in acetonitrile). Data were processed using Xcalibur.4.4 (Thermo Fisher Scientific). Cytokinins (CKs) were measured by UPLC equipped with a XevoTQ-XS Triple Quadrupole MS (Waters) using an ODS column with linear gradients of solvent A (0.06% acetic acid) and B (0.06% acetic acid in 70% methanol). Data were processed using MassLynx 4.1 (Waters).

### DESI–MSI analysis

Cryosections of flower buds of the WT and *Sldad1* mutants at −2, −1, and 0 DAA were created and analysed via desorption electrospray ionization–MS imaging (DESI–MSI) according to a previously described method (Enomoto, 2021) with minor modifications. Briefly, the fresh flower buds were immersed in 2% carboxymethyl cellulose (CMC) and immediately frozen in liquid nitrogen. An adhesive film was then attached to the cross-section, and cryosections (30 μm) were created using a cryostat (CM 3050; Leica Microsystems). The film was then attached to Matsunami adhesive silane (MAS)-coated glass slides (Matsunami Glass Inc.) with adhesive tape, placed in 50 ml conical tubes containing silica gel for drying, and preserved at −80 °C until DESI–MSI analysis.

A Waters SYNAPT XS HDMS DESI–MSI system was used, and the data were acquired with a 100 μm or 200 μm step size in the negative ion and sensitivity modes at 100–300 *m*/*z*. The DESI solvent of 1 mM ammonium acetate in 95% aqueous methanol was injected, and *m*/*z* values were calibrated externally using 10 mmol l^–1^ sodium formate solution in 90% aqueous 2-propanol. The acquired mass spectra were calibrated internally using the *m*/*z* of the palmitic acid [M-H]^–^ ion (*m*/*z* 255.2330), normalized by total ion current. The data were processed and visualized with the Waters High Definition Imaging (HDI) software v1.5. Ion images were obtained by JA [M-H]^–^ ion (exact *m*/*z* 209.1183). Three flowers were analysed, each from a different plant.

### Immunocytological analysis

Ovaries were fixed with 4% (w/v) 1-ethyl-3-(3-dimethyl aminopropyl)-carbodiimide hydrochloride (ThermoFisher Scientific, 22981) in phosphate-buffered saline (PBS), embedded in polyethylene glycol (average molecular weight 1500), and incubated with an anti-JA antibody detecting both JA and JA-Ile (Agrisera, AS11 1799) at 1:500 in PBS containing 5% (w/v) bovine serum albumin (BSA) overnight at 4 °C as described previously (Mielke *et al.*, 2011) with minor modification. After three washes in PBS/0.1% BSA and one wash in PBS/1% BSA, sections were incubated with the secondary antibody goat-anti-rabbit-IgG AlexaFluor488 (Abcam, ab150077, 1:500 diluted) for 2 h at 37 °C. After four washes with PBS, sections were mounted in Prolong Gold Mountant (ThermoFisher Scientific, P36966). The negative control was not treated with the anti-JA antibody. Fluorescence images were observed and analysed using the Zeiss LSM700 microscope and ZEN 2009 software.

### Exogenous treatment with a GA inhibitor

Flowers were removed so that only one non-emasculated flower remained per plant. At 0 DAA, the WT and *Sldad1* flowers were sprayed with 50 μM paclobutrazol (PAC; Sigma-Aldrich, 43900) dissolved in 1% ethanol containing 0.1% Tween 20. For the mock treatment, flowers were sprayed with the same solution without PAC. Ovary diameters were measured at 10 DAA, as described above.

### Sequencing analysis

Genomic DNA was extracted from 3-week-old ‘Micro-Tom’ seedlings using a DNeasy Plant Mini Kit (Qiagen, 69104). The PCR products were purified using a FastGene Gel/PCR Extraction Kit (Nippon Genetics, FG-91302), the genomic sequence of *SlDAD1* was determined using the extracted DNA and gene-specific primers (Supplementary Table S1).

### Transcriptome sequencing and data analysis

Total RNA was extracted from three biological replicates of filaments at −2 DAA and 0 DAA and of ovaries from −2 DAA to 4 DAA using RNeasy Plant Mini Kits (Qiagen, 74904), and purified using the RNase-Free DNase Set (Qiagen, 79254). RNA-seq data with a quality score of <30 were discarded; adapters were trimmed using TrimGalore (v.0.6.7) with the option -length 90, and rRNA reads were filtered using SortMeRNA (v.2.1). The quality of the clean reads obtained from RNA-seq was assessed with FastQC (v.0.11.3). The cleaned reads were mapped to the tomato reference genomes SGN release SL4.0 (https://solgenomics.net/), and the transcript levels were calculated using the STAR (v.2.5.3a)–RSEM (v.2.7.10a) pipeline. Read counts per gene were expressed as transcripts per million mapped reads (TPM>0). Read counts were generated using featureCounts (v.1.6.4). Differentially expressed genes (DEGs) were identified using the R package DEseq2 (v1.42.1) (Love *et al.*, 2014) according to the thresholds absolute fold-change (FC) >1 and false-discovery rate (FDR) <0.05. Gene annotations were assigned according to the ITAG 4.0 loci descriptions (https://solgenomics.net/ftp/tomato_genome/annotation/ITAG4.0_release/ITAG4.0_descriptions.txt). Gene Ontology (GO) terms were obtained from the PANTHER classification system v.10 (Mi *et al.*, 2016). GO enrichment analysis was performed for ‘GO biological process complete’ using the Bioconductor R library clusterProfilar (v.4.10.1) with FDR correction (adjusted *P*-value<0.05) (Yu *et al.*, 2012). The *z*-score was calculated for each gene per sample using the formula (*X*−*X*_av_)/*X*_SD_, where *X* is the TPM value (raw read count data+0.01), *X*_av_ is the mean of TPM values, and *X*_SD_ is the standard deviation of TPM values. Heatmaps were created using the R package pheatmap (v.1.0.12).

### Statistical analysis

All statistical analyses were performed using R (v.4.3.2). For comparisons between two groups, significant differences were evaluated using Welch’s *t*-test or Mann–Whitney *U* tests, and for comparisons among multiple groups differences were evaluated using ANOVA followed by the Tukey–Kramer test.

## Results

### Phenotypes of the novel seedless *Sldad1* mutant

**Fig. 1.**
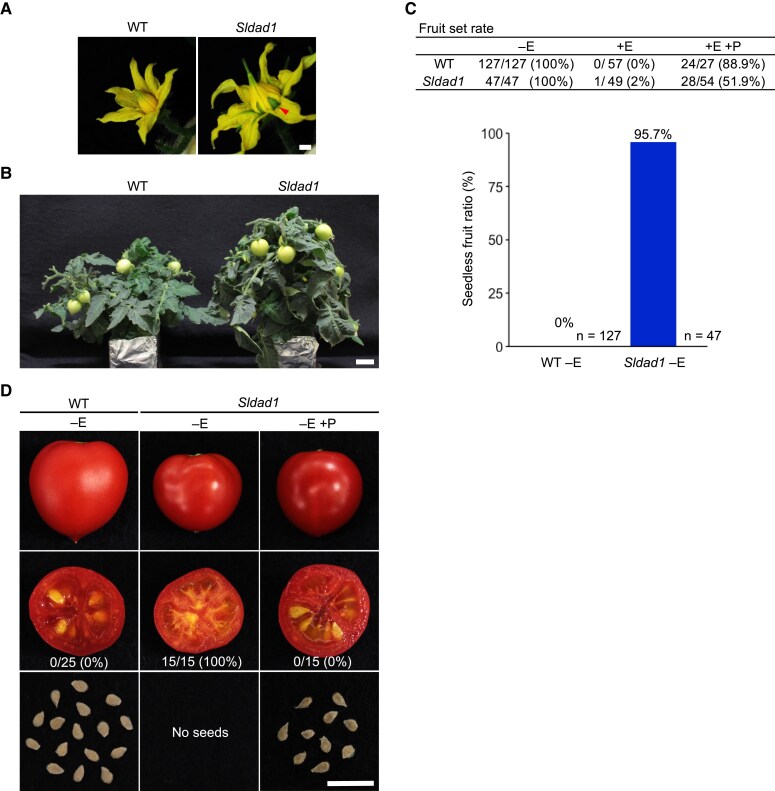
Phenotypes of the tomato ‘Micro-Tom’ wild type (WT) and *Sldad1* mutant. (A) Representative images of the flowers at 4 days after anthesis (DAA). The red arrowhead indicates dehiscence at the base of the anther cone caused by early ovary enlargement. Scale bar is 2 mm. (B) Representative images of 40-day-old WT and *Sldad1* mutant plants. Scale bar is 2 cm. (C) Fruit set rate and seedless fruit ratio of non-emasculated (–E) and emasculated (+E) flowers of the WT and *Sldad1* flowers. Self-pollination (+P) was performed manually after emasculation. The number of fruits set together with the number of ovaries monitored are shown. (D) Images of fruit and their seeds at the red-fruit stage of the WT and *Sldad1* mutant. The mutant was self-pollinated at 0 DAA. Scale bar is 1 cm. The numbers of seedless fruits and the total number of fruits examined are shown, together with their ratio. Fruits were harvested from at least five plants.

To investigate the molecular mechanisms underlying tomato fruit set, we examined a previously published large ‘Micro-Tom’ EMS-mutagenized population (Saito *et al.*, 2011) for mutants showing anther cone dehiscence by early ovary enlargement, and selected one seedless mutant, which was designated as *Solanum lycopersicum*  *defective in anther dehiscence 1* (*Sldad1*) (Fig. 1A, B).

To assess the degree of seedlessness, we performed an emasculation experiment. As determined at the red-fruit stage, none of the emasculated WT flowers showed fruit formation, whereas all non-emasculated WT flowers produced fruits (Fig. 1C; Supplementary Fig. S1A). A high ratio of seedless fruit was observed in non-emasculated *Sldad1* fruits (95.7%) but unexpectedly, the fruit set rate was decreased to 2% when the anther was emasculated. When manually self-pollinated after emasculation, the fruit set rate of emasculated *Sldad1* flowers was still decreased, to ∼50%, suggesting that emasculation negatively affected fruit set even in pollinated flowers. Additionally, pollen grains on the stigma of non-emasculated *Sldad1* flowers were not present even after anthesis (Supplementary Fig. S1B, C), and the developed fruits were almost all seedless (Fig. 1C). These results suggested that emasculation probably one of the causes of the lack of fruit set in the *Sldad1* mutant.

The overall phenotype of *Sldad1* plants was similar to the WT, except for a slight increase in height (Fig. 1B). We evaluated the fruit phenotypes, including length, diameter, columella weight, pericarp weight, 100-seed weight, and locule number (Supplementary Fig. S1D). Given that pollen grains were observed on the stigma surface of WT flowers after anthesis but not on those of the *Sldad1* mutant, we emasculated WT flowers in this study to prevent pollination. The size of the non-emasculated *Sldad1* fruits did not differ from that of the WT (Fig. 1D; Supplementary Fig. S1D), and we did not observe any unexpected phenotypes such as fruit morphological abnormalities in the mutant. In terms of male reproductive phenotypes, the mutant was similar to the WT in anther structure, anther dehiscence, pollen grain number, and pollen germination ratio on both the stigma on medium (Supplementary Fig. S2A–E). In *Sldad1*, stigma exsertion of 0.5–1 mm was observed from 0 DAA in all flowers (Supplementary Fig. S3A, B), suggesting that a failure in self-pollination caused by stigma exsertion was the likely reason why most of the spontaneously produced *Sldad1* fruits were seedless, whilst *Sldad1* fruits successfully produced seeds via manual pollination (Fig. 1D). This stigma exsertion beyond the anther cone might be driven by ovary growth.

### Identification of the gene responsible for the *Sldad1* mutant

To investigate the inheritance pattern of the mutant, we crossed *Sldad1* plants with ‘Micro-Tom’ WT and ‘Resina’. The phenotypes of the F_2_ offspring segregated in a WT:mutant phenotype ratio of 156:48 (χ^2^=0.63; Supplementary Fig. S4), indicating that the *Sldad1* mutation is monogenic recessive.

**Fig. 2.**
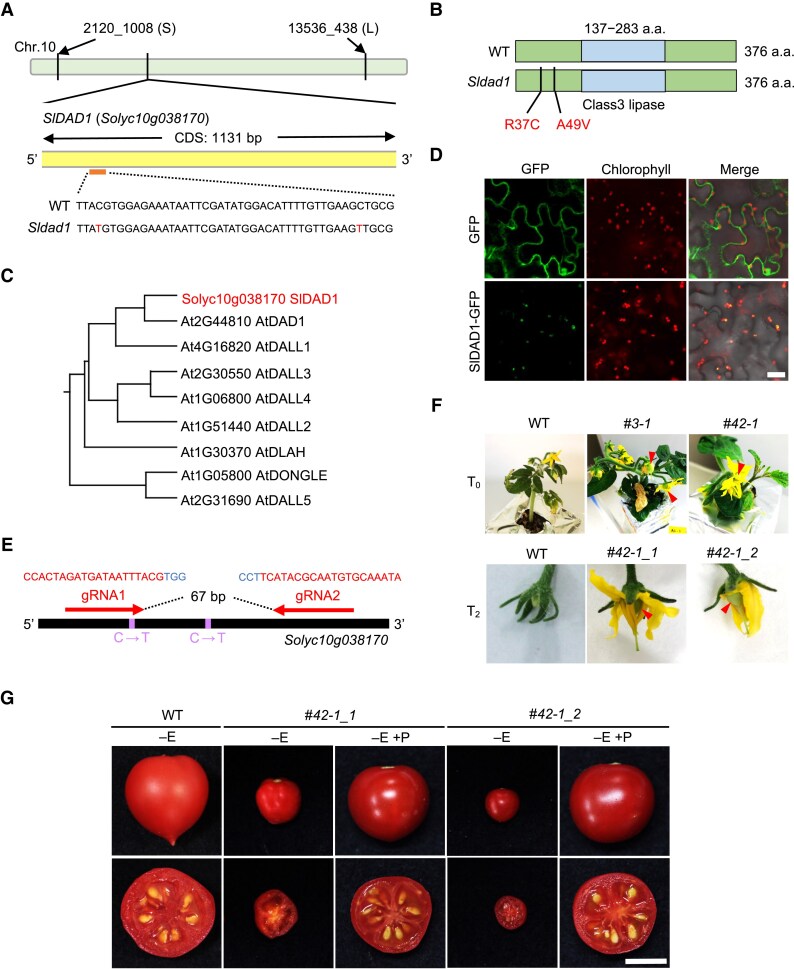
Identification of the candidate gene for the *Sldad1* mutant and functional analysis of the DAD1 protein. (A) Location and gene structure of *SlDAD1*, and the mutation present in *Sldad1*. CDS, coding sequence; WT, wild type; a.a., amino acids. (B) Putative SlDAD1 protein as predicted by Pfam, together with the *Sldad1* mutated form with two amino acid missense mutations. (C) Phylogenetic tree of SlDAD1 and its homologous proteins in Arabidopsis, as determined using CLUSTALW. (D) Subcellular localization of the tomato DAD1 protein. SlDAD1 was fused to green fluorescence protein (GFP) and transiently expressed in *Nicotiana benthamiana* leaves. Auto-fluorescence images of chlorophyll are also shown together with merged images. An empty vector with GFP alone was used as a negative control. The images are representative of three biological replicates. Scale bar is 20 μm. (E) Target sites of gRNAs for CRISPR/Cas9 editing in the tomato ‘Micro-Tom’ background. Blue indicates PAM sequences. (F) Phenotypes of the *SlDAD1*-knockout lines resulting from CRISPR/Cas9 editing. The T_0_ plants *#3-1* and *#42-1* were homozygous and biallelic, respectively. T_2_ plants were obtained by self-pollination, and two independent genome editing patterns were obtained. Red arrowheads indicate early ovary development. (G) Phenotypes of fruits at the red stage of the WT and CRISPR/Cas9 mutants. Scale bar is 1 cm. None of the plants were emasculated (–E). +P indicates that self-pollination was performed manually after emasculation. Seedless fruits were formed in the CRISPR/Cas9 mutants without manual pollination.

To identify the gene responsible for the mutant, we employed rough mapping to narrow down the region of the causative gene. Twelve F_2_ seedless populations generated by crossing *Sldad1* and ‘Resina’ and 24 single-nucleotide polymorphism (SNP) markers were used for genotype mapping. Rough mapping indicated that the candidate gene locus was located on the short arm of chromosome 10, based on SNP markers between 2120_1008 (S) and 13536_438 (L) (Fig. 2A; Supplementary Fig. S5).

Next, we performed whole-genome re-sequencing of BC_1_S_1_, BC_2_S_1_, and BC_3_S_1_ populations derived from the *Sldad1* mutant using Illumina HiSeq X Ten and HiSeq 2000 next-generation sequencing. Using BC_3_F_2_ and BC_2_F_2_, we aligned the cleaned reads to the tomato reference genome SL2.50 or SL3.0 (Tomato Genome Consortium, 2012) and identified SNPs specific to the *Sldad1* mutant. Four candidate genes, *Solyc10g009590*, *Solyc10g009640*, *Solyc10g019033*, and *Solyc10g038170*, were selected for further linkage analysis based on their relatively high expression levels in flowers and fruits, and the presence of mutations within exons (Supplementary Fig. S6A, B). Linkage analysis identified 21 seedless plants and 32 plants with seeds in the BC_3_S_1_ population (Supplementary Fig. S6C). Moreover, RPKM values indicated that *Solyc10g038170* was expressed in the ovaries, while *Solyc10g019033* was not detected (Supplementary Fig. S6D), suggesting *Solyc10g038170* as the most likely candidate gene of the novel seedless mutant.

According to the Solonaceae Genomics Network, *Solyc10g038170* is a 1131 bp gene consisting of a single exon. It encodes a conserved domain of the Class 3 lipase, specifically a triglyceride lipase, as predicted by Pfam, although two mutated regions were not included in this domain. The *Sldad1* mutant has two single-nucleotide substitutions at the 109th and 146th bases from the start of the exon (C>T), resulting in two amino acid missense mutations, R37C and A49V, respectively (Fig. 2B). NCBI BLAST searches against protein databases identified eight related protein family members. Multiple-sequence alignment analysis showed that the protein sequence of the candidate gene has 63% identity with DAD1 in Arabidopsis, and a phylogenetic tree constructed from tomato and Arabidopsis indicated that *Solyc10g038170* is most homologous to *AtDAD1* (Fig. 2C). *AtDAD1* encodes PHOSPHOLIPASE A1 that is released in the filament and required for α-LA release in the first step of the JA biosynthesis pathway (Ishiguro *et al.*, 2001). TargetP v1.1 (https://services.healthtech.dtu.dk/services/TargetP-1.1/) predicted chloroplastic localization for DAD1, and we investigated the subcellular localization of tomato DAD1 using confocal microscopy. GFP was fused to SlDAD1, with the p19 empty vector as the negative control. Transient expression of SlDAD1*-*GFP in *N. benthamiana* leaves confirmed plastid localization (Fig. 2D), consistent with a previous report for DAD1 in Arabidopsis (Ishiguro *et al.*, 2001). A conserved arginine at the 37th position, shared between *SlDAD1* and *AtDAD1* (Supplementary Fig. S7), suggests that this site might be more critical for function than the A49V mutation. We next examined the fruit set phenotype of the identified mutation for a cross with another tomato variety by introducing it into the ‘Ueleie 106 WP’ background. In the resulting F_3_ population, individuals homozygous for the *Sldad1* allele that were emasculated exhibited high fruit set, whereas those carrying the WT allele or the heterozygous genotype showed low fruit set (Supplementary Fig. S6E). Moreover, the *Sldad1* allele with emasculation exhibit potentially high yield under heat-stress conditions (Supplementary Fig. S6F), consistent with the trend observed in the ‘Micro-Tom’ background (Supplementary Fig. S6G, H). These results supported the idea that the selected mutation is probably responsible for the seedless phenotype.

To further validate that *Solyc10g038170* is the gene responsible for the *Sldad1* mutant phenotype, we generated knockout plants using the CRISPR/Cas9 genome-editing system. We designed two target sites within *SlDAD1* (Fig. 2E) and obtained six independent T_0_ lines. The homozygous and bi-allelic T_0_ lines exhibited early ovary enlargement and the persistence of petals and styles after ovary enlargement, similar to those found in the *Sldad1* mutant (Fig. 2F; Supplementary Fig. S8AC). Although we did not obtain multiple T_2_ lines, we identified one homozygous T_2_ line with two distinct editing patterns (#*42*-*1*_*1* and #*42*-*1*_*2*), and both were seedless (Fig. 2G; Supplementary Fig. S8D). In addition, similar phenotypes were observed in both the *Sldad1* and CRISPR/Cas9 mutants, such as style protrusion, browning of anther cones, and failure of ovary enlargement in terminal inflorescences, (Fig. 2F; Supplementary Fig. S8E, F). Thus, we concluded that *Solyc10g038170* is the gene responsible for the novel seedless mutant *Sldad1*.

### Spatiotemporal expression of *SlDAD1* and JA concentrations

The JA-deficient mutant *jai1-1* has previously been reported to exhibit a delay in flower development (Niwa *et al.*, 2018). We found that the *Sldad1* mutant also showed a minor delay, with flower development from 5 mm buds to anthesis taking an average of 5.7 d compared with 4.8 d in WT plants (Supplementary Fig. S3A, C).

**Fig. 3.**
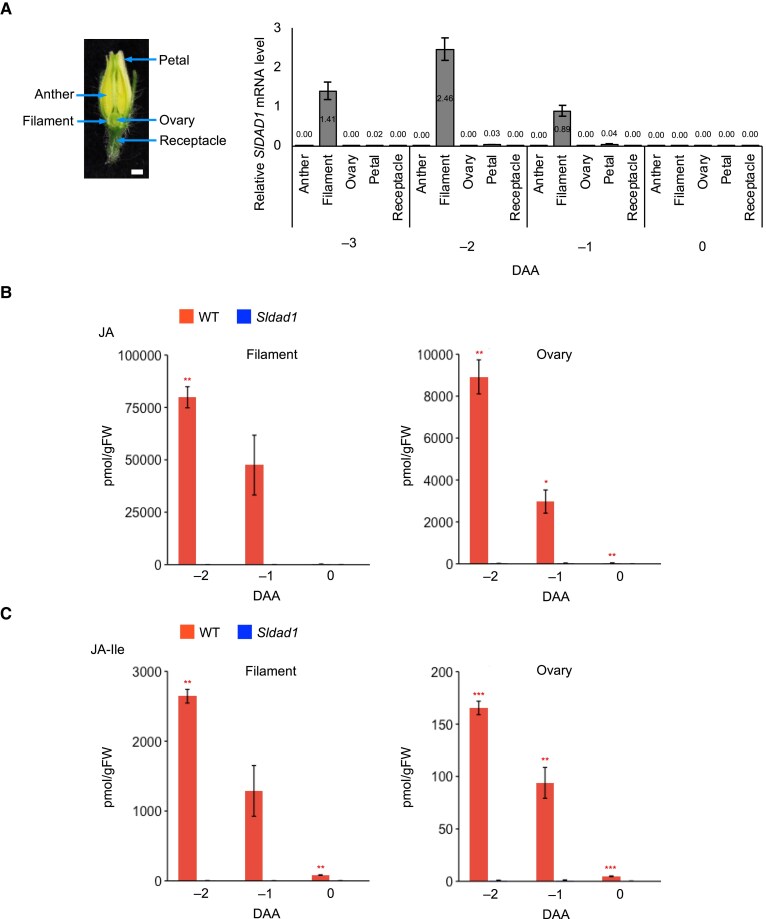
Tissue-specific expression of *SlDAD1* in tomato floral buds and endogenous jasmonate levels in filaments and ovaries. (A) Relative *SlDAD1* transcript levels in different floral tissues of the ‘Micro-Tom’ wild type, as determined by qRT-PCR analysis using *SAND* as the internal control. DAA, days after anthesis. The scale bar in the image is 1 mm. (B, C) Concentrations of (B) jasmonic acid (JA) and (C) JA-isoleucine (JA-Ile) in the filaments and ovaries of the wild type (WT) and the *Sldad1* mutant. All data are means (±SE), *n*=4. Significant differences compared with the WT were determined using Welch’s *t*-test: *P<0.05, **P<0.01, ***P<0.001.

To explore the expression patterns of *SlDAD1*, we analysed mRNA levels. *In silico* analysis using the tomato eFP browser (http://bar.utoronto.ca/eplant_tomato/) indicated high expression in unopened flower buds (Supplementary Fig. S3D). qRT-PCR analysis from −5 DAA to 0 DAA confirmed that *SlDAD1* was highly expressed in WT buds before anthesis, peaking at −2 DAA and then markedly declining by 0 DAA (Supplementary Fig. S3E). Further analysis across anthers, filaments, ovaries, petals, and receptacles from −3 DAA to 0 DAA, revealed that *SlDAD1* transcripts predominantly accumulated in WT filaments before anthesis, especially at −2 DAA, whereas expression was very low or undetectable in the other tissues (Fig. 3A). *SlDAD1* transcripts tended to be induced in filaments and ovaries of the *Sldad1* mutant following anther removal (Supplementary Fig. S3F, G), similar to the wound-induced expression of *AtDAD1* previously reported (Ishiguro *et al*., 2001).

To assess JA concentrations in female reproductive organs, we sampled flower buds of the WT and *Sldad1* mutant from −3 DAA to 0 DAA. JA and JA-Ile concentrations in the WT plants peaked at −2 DAA (Supplementary Fig. S3H, I). We then collected filaments and ovaries at −2, −1, and 0 DAA and found that in the WT, the JA and JA-Ile concentrations were high before anthesis, particularly at −2 DAA, and subsequently declined as flowering progressed, reaching nearly zero after 0 DAA (Fig. 3B, C). Notably, endogenous JA and JA-Ile accumulated in both the WT filaments and ovaries before anthesis. In contrast, in the *Sldad1* mutant almost no JA or JA-Ile accumulation was detected in the floral organs at any developmental stage (Fig. 3B, C; Supplementary Fig. S3H, I). This observation aligns with previous findings for the carpeloid *tap3* mutant, which carries mutations in the B-class tomato MADS-box gene *APETALA3* and exhibits significantly reduced JA concentration at −2 DAA compared to the WT (Kusano *et al.*, 2022) (Supplementary Fig. S9A, B). The changes in the JA and JA-Ile concentrations correlated closely with the trend of *SlDAD1* expression trend, suggesting that the JA synthesized in the filament is transported to the ovary before anthesis, suppressing the fruit set.

### JA is localized in the filament and ovule

**Fig. 4.**
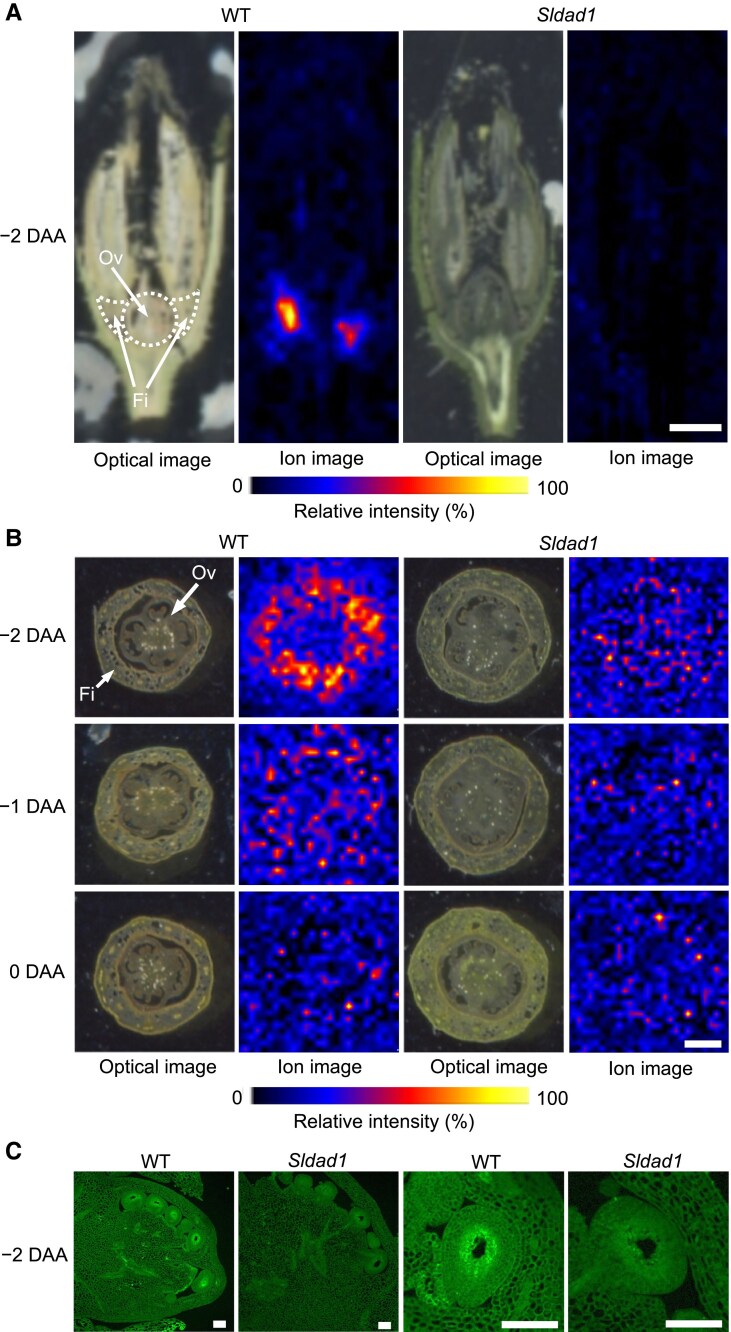
Visualization of jasmonates in the floral organs of the tomato ‘Micro-Tom’ wild type (WT) and the *Sldad1* mutant. (A) Optical and ion images of the distribution of jasmonic acid (JA) obtained by DESI–MSI of longitudinal sections of floral buds at −2 days after anthesis (DAA). The images are representative of three biological replicates; scale bar is 2 mm. Fi, filament; Ov, ovary. The data were acquired with a 200 μm step size in the negative ion. Internal calibration of the MS/MS spectrum was performed post-acquisition using the exact *m/z* of JA (209.12). (B) Optical and ion images of transverse sections of floral buds at −2, −1, and 0 DAA. The images are representative of three biological replicates; scale bar is 0.5 mm. The data were acquired with a 100 μm step size in the negative ion. (C) Immunocytochemical detection of JA and JA-isoleucine (JA-Ile) in ovaries and ovules at −2 DAA. Scale bars are 100 μm. JA/JA-Ile were detected using an anti-JA antibody and are visualized by green fluorescence.

To test the hypothesis that JA synthesized in the filament is transported to the ovaries, we examined its localization in floral organs prior to anthesis using DESI–MSI, a powerful technique for visualizing metabolites in plant tissues, including seeds (e.g. Enomoto *et al.*, 2017; Enomoto, 2021). Our analysis of tomato bud sections showed that JA accumulated more in the filaments than in the ovaries before anthesis, particularly at −2 DAA (Fig. 4A, B). It was barely detected in the ovaries, probably because its concentration was only about one-tenth of that in the filaments, and DESI–MSI analysis depends on the pixel with the highest signal intensity in the image. We further investigated JA localization in the ovaries using immunocytological analysis. The signals for JA/JA-Ile detected by the antibody in the WT ovaries were stronger than in the *Sldad1* mutant at −2 DAA (Fig. 4C), consistent with the results for JA and JA-Ile concentrations (Fig. 3B, C). Notably, JA and JA-Ile were observed to accumulate in the ovules, specifically around the embryo sac. Additionally, differentiation of endothelial cell was observed around the embryo sac in the *Sldad1* ovules (Supplementary Fig. S2F), where JA/JA-Ile localized in the WT ovules, as detected by immunohistochemical staining. These findings supported the hypothesis, and suggested that the high level of accumulation of JA and JA-Ile in the ovule integument is particularly important for suppressing ovary development before anthesis.

### *Sldad1*-induced ovary growth is primarily promoted by cell expansion

**Fig. 5.**
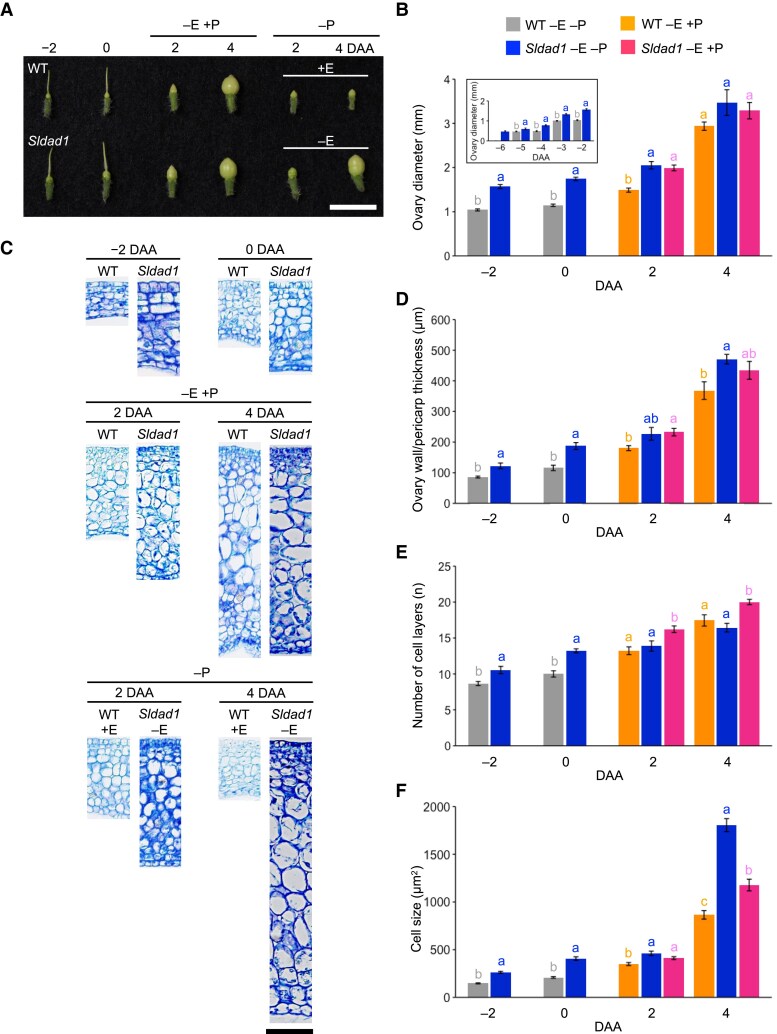
Ovary development in the tomato ‘Micro-Tom’ wild type (WT) and the *Sldad1* mutant. (A) Representative images of WT and *Sldad1* ovaries from −2 days after anthesis (DAA) to 4 DAA. Scale bar is 1 cm. −E, non-emasculated; +E, emasculated; −P, unpollinated; +P, manually pollinated. (B) Ovary diameter. The inset shows the diameter during early ovary development. (C) Representative images of cross-sections and (D) ovary wall/pericarp thickness from −2 DAA to 4 DAA. Scale bar is 100 μm. (E) Number of cell layers and (F) cell size in the ovary wall. All data are means (±SE), *n*=5–10 for –6 DAA to –3 DAA, and *n*=10 for –2 DAA to 4 DAA. Different letters indicate significant differences among means at each time-point as determined using ANOVA followed by Tukey–Kramer tests (*P<0.05).

To better understand the phenotype of the *Sldad1* mutant, we measured ovary diameter during early development. The diameter of *Sldad1* ovaries in non-emasculated plants was significantly greater than that of the WT from –2 DAA to 2 DAA, and the mutant ovaries showed marked enlargement from 2 DAA to 4 DAA, becoming comparable in size to those of the pollinated WT (Fig. 5A, B).

To investigate whether this growth was due to cell division or expansion, we examined cross-sections of ovaries from −2 DAA to 4 DAA. Between −2 DAA and 0 DAA, the ovary wall thickness, number of cell layers, and cell area of the ovary wall were greater in non-emasculated *Sldad1* ovaries than in the WT (Fig. 5C–F). Following pollination, both WT and *Sldad1* ovaries displayed increased wall thickness due to additional cell layers and enlarged cells. At 2 DAA to 4 DAA, non-emasculated *Sldad1* ovaries had a significantly greater wall thickness than the WT, mainly driven by a greater increase of cell areas within the wall, accompanied by an increase in the number of cell layers. Thus, the greater ovary wall thickness observed in non-emasculated *Sldad1* ovaries was primarily due to enhanced cell expansion rather than increased division.

### *Sldad1* seedless fruits exhibit increased GA levels

**Fig. 6.**
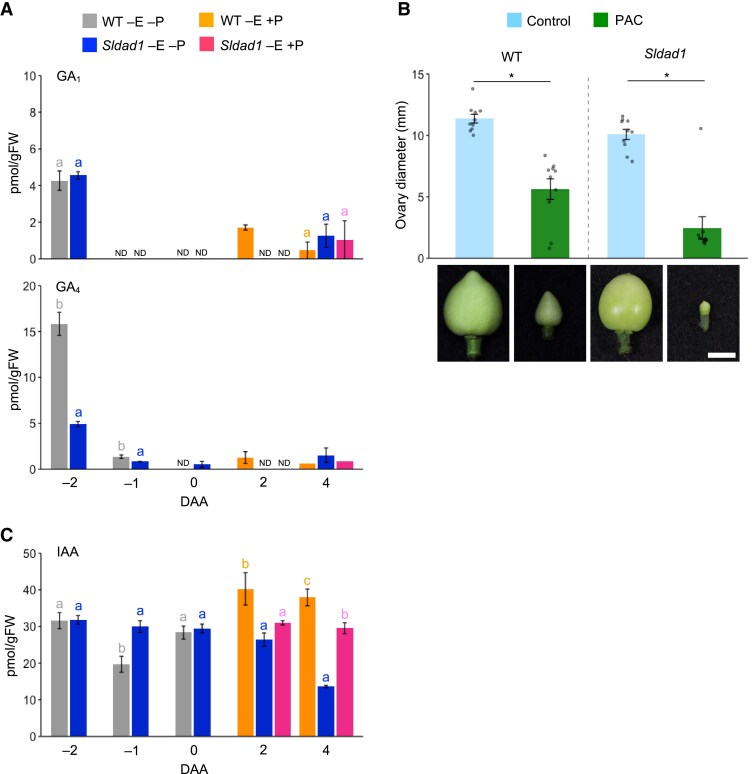
Endogenous levels of bioactive gibberellins (GAs) and auxin (IAA) in the ovaries of the tomato ‘Micro-Tom’ wild type (WT) and the *Sldad1* mutant. (A) Concentrations of bioactive GAs (GA_1_, GA_4_) in the ovaries of non-emasculated (–E) flowers either with (+P) or without (–P) self-pollination performed manually after emasculation. DAA, days after anthesis; ND, not detected. (B) The effect of the GA biosynthesis inhibitor paclobutrazol (PAC) on ovary development. Flowers were sprayed with either 50 μM PAC or a control solution at 0 DAA and ovary diameter was measured at 10 DAA. Representative images of ovaries are shown; scale bar is 5 mm. Data are mean (±SE), *n*=10. Significant differences were determined using Mann–Whitney *U* tests: *P<0.05. (C) Concentrations of indole-3-acetic acid (IAA) in the ovaries. Data in (A, C) are mean (±SE), *n*=3–4. Different letters indicate significant differences among means at each time-point as determined using ANOVA followed by Tukey–Kramer tests (*P<0.05).

The increased cell expansion in the *Sldad1* mutant suggested that GAs might be responsible for inducing ovary enlargement, and hence we measured GA concentrations in the ovaries. Two parallel GA biosynthesis pathways operate, namely the early-13-hydroxylation and non-13-hydroxylation pathways, which produce bioactive GA_1_ and GA_4_, respectively (Supplementary Fig. S10A). We assayed these two bioactive GAs as well as precursors (GA_12_, GA_24_, GA_53_, GA_44_, GA_19_, and GA_20_) and a catabolite (GA_8_). We found that GA_1_ and GA_4_ were undetectable in non-emasculated *Sldad1* ovaries at 2 DAA but they were present at 4 DAA (Fig. 6A). The GA_1_ precursors GA_53_, GA_44_, GA_19_, and GA_20_ and the catabolite GA_8_ remained detectable or showed increases at 2 DAA and 4 DAA (Supplementary Fig. S10B, C), suggesting that metabolism via the early-13-hydroxylation pathway was activated in response to the reduced JA biosynthesis. Before anthesis, GA_4_ concentrations were significantly lower in *Sldad1* ovaries than the WT, whereas GA_1_ levels were similar (Fig. 6A).

To investigate whether GAs influenced fruit set in *Sldad1*, we applied the GA-biosynthesis inhibitor PAC directly to non-emasculated *Sldad1* ovaries at 0 DAA. Out of 10 *Sldad1* ovaries treated with 50 μM PAC, nine exhibited substantially lower growth compared to the mock treatment at 10 DAA (Fig. 6B), indicating that GA biosynthesis was required for fruit set in non-emasculated *Sldad1*.

To determine whether fruit formation in the *Sldad1* mutant correlated with increased auxin concentrations, we measured IAA during ovary development. In pollinated WT ovaries, IAA concentrations were significantly increased at 2 DAA and 4 DAA, whereas in pollinated *Sldad1* ovaries the concentrations IAA remained unchanged throughout all the developmental stages examined (Fig. 6C). In non-emasculated *Sldad1* ovaries, the concentration had declined at 4 DAA. In addition, the IAA concentration in *Sldad1* ovaries was higher than in the WT at −1 DAA, suggesting that auxin biosynthesis was enhanced by the mutation before anthesis.

Fruit set is regulated by a complex network involving multiple phytohormones, including CKs, ABA, and ethylene. We examined the concentrations of the bioactive CKs *trans*-zeatin (*t*Z), *t*Z riboside (*t*ZR), *N*^6^-(Δ^2^-isopentenyl) adenine (iP), and *N*^6^-(Δ^2^-isopentenyl) adenosine (iPR), and found that concentrations of iP were consistently high in non-emasculated *Sldad1* ovaries (Supplementary Fig. S11A). ABA concentrations were high until anthesis in both the WT and *Sldad1* mutant, and declined following pollination (Supplementary Fig. S11B); however, the concentrations were higher in non-emasculated *Sldad1* ovaries compared with pollinated ovaries.

### Transcriptome analysis of phytohormone-related genes altered in the *Sldad1* mutant

**Fig. 7.**
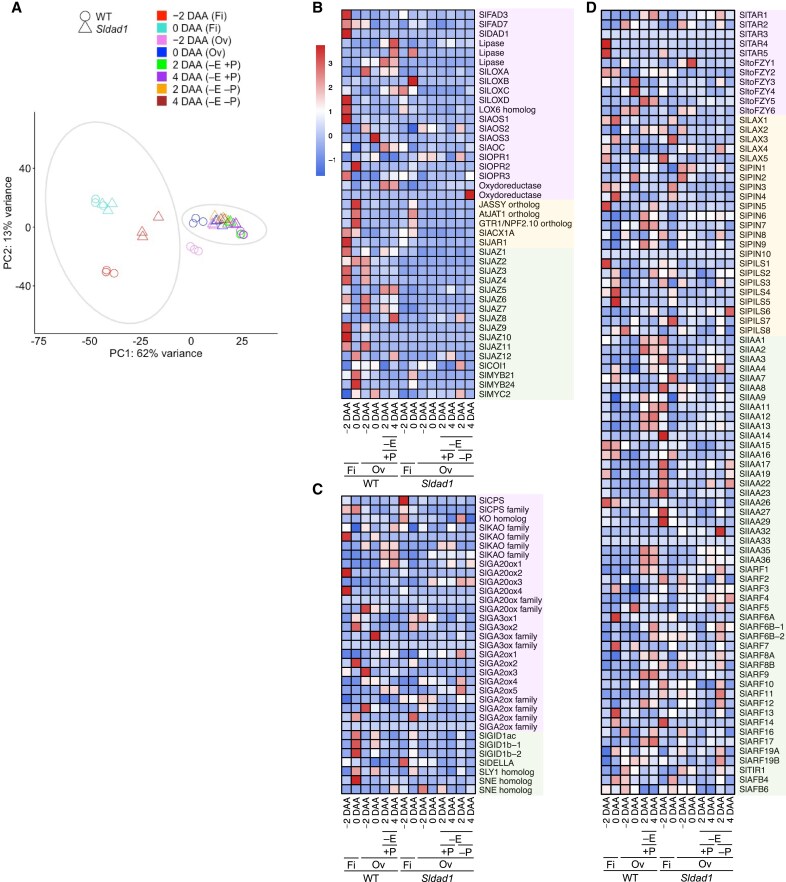
Gene expression profiles of the filaments and ovaries of the tomato ‘Micro-Tom’ wild type (WT) and the *Sldad1*. (A) Principal component analysis of RNA-seq results for filaments (Fi) at –2 days after anthesis (DAA) and 0 DAA, and ovaries (Ov) at –2, 0, 2, and 4 DAA in non-emasculated (–E) flowers either with (+P) or without (–P) self-pollination performed manually after emasculation. (B–D) Heatmaps showing the transcript levels of genes related to (B) jasmonic acid, (C) gibberellins, and (D) auxin at different time-points in the filaments and ovaries, based on transcripts per million and normalized expression values converted to *z*-scores. Pink, yellow, and green shading of the gene names indicate their involvement in biosynthesis, transport, and signaling, respectively.

To gain insights into how JA biosynthesis regulates tomato fruit set, RNA-seq analysis was conducted on WT and *Sldad1* filaments and ovaries at −2, 0, 2, and 4 DAA. Gene expression at −2, −1, and 0 DAA was validated using qRT-PCR in an independent experiment (Supplementary Fig. S12). Principal component analysis showed that the transcriptomes clustered distinctly by tissue type and developmental stage, with PC1 (62%) separating based on tissue (filament and ovary) and PC2 (13%) according to flowering stage (Fig. 7A). Differentially expressed genes (DEGs) related to fruit set were identified between the WT and *Sldad1* during early ovary development. Compared to the WT, *Sldad1* ovaries at −2 DAA (corresponding with the JA and JA-Ile concentration peaks) displayed 2538 DEGs, including 1172 up- and 1366 down-regulated genes [|log_2_ (fold-change)|>1, FDR<0.05) (Supplementary Fig. S13; Supplementary Tables S2, S3). GO terms for the down-regulated DEGs in the *Sldad1* ovaries were enriched for pathways including ‘regulation of jasmonic acid mediated signaling pathway’ at −2 DAA (Supplementary Fig. S14A). On the other hand, up-regulated DEGs in the *Sldad1* ovaries included terms related to cell division, such as ‘microtubule-based movement’ and ‘regulation of mitotic spindle organization’ at −2 DAA and 0 DAA (Supplementary Fig. S14B), aligning with the increases in ovary growth and number of cell layers observed in the non-emasculated *Sldad1* mutant (Fig. 5). These results suggested that activation of cell division pathways also contributed to ovary development in the mutant.

We further investigated the expression patterns of genes related to JA biosynthesis, transport, and signaling in the filament and ovary using RNA-seq. Among the biosynthesis genes, *SlDAD1*, *SlAOS1*, and *SlOPR3* were highly expressed in WT filaments at −2 DAA (Fig. 7B). In contrast, the expression of JA biosynthesis and signaling genes remained low in both filaments and ovaries of *Sldad1*. The downstream JA signaling gene *SlJAZ9*, encoding a protein known to interact with the JA-related transcription factor SlMYB21, was highly expressed in WT filaments and ovaries at −2 DAA (Fig. 7B). JA biosynthesis is activated by the degradation of SlJAZ9, releasing SlMYB21 (Schubert *et al.*, 2019). The expression patterns of the JA biosynthesis and signaling genes aligns with the reduced accumulation of JA and JA-Ile in the *Sldad1* mutant. Consistent with previous findings in the *jai1-1* mutant (Schubert *et al.*, 2019), *SlMYB21* was significantly down-regulated in the *Sldad1* ovaries at −2 DAA and 0 DAA, while its homolog *SlMYB24* showed no significant differences between the WT and *Sldad1* (Fig. 7B; Supplementary Table S3). Additionally, the tomato orthologs *Solyc01g008010*, *Solyc05g054890*, and *Solyc03g113420* of known JA transporters in Arabidopsis such as *JAT* (Li *et al.*, 2020), *JASSY* (Guan *et al.*, 2019), and *GTR/NPF2.10* (Saito *et al.*, 2015), were up-regulated in WT filaments compared with *Sldad1*. Taken together, these findings support the dynamic role of JA in tomato floral organ development.

The increased GA accumulation observed in non-emasculated *Sldad1* ovaries suggested that JA might influence tomato fruit set through GA pathways. To investigate whether the elevated concentrations of bioactive GAs resulted from transcriptional regulation, we compared the expression patterns of four GA20-oxidase (*GA20ox*) and two GA3-oxidase (*GA3ox*) GA-biosynthesis genes, and of five GA2-oxidase (*GA2ox*) GA-inactivation genes in the WT and *Sldad1* mutant. In non-emasculated *Sldad1* ovaries at 4 DAA, the expression levels of *SlGA20ox1* and *SlGA20ox3* increased, while *SlGA2ox4* and *SlGA2ox5* were down-regulated (Fig. 7C), whilst transcript levels of *SlGA3ox1* and *SlGA3ox2* remained unchanged. These findings suggested that JA negatively regulates GA biosynthesis while promoting GA inactivation.

We also examined the involvement of JA biosynthesis in regulating auxin-related genes. Genes encoding AUX/IAA proteins and auxin response factors (ARFs) were up-regulated in *Sldad1* ovaries. *SlARF5* and *SlARF7*, which are primarily located in the pericarp and ovules of tomato, act as repressors of fruit set, with *slarf5* and *slarf7* mutants exhibiting parthenocarpy (de Jong *et al.*, 2009; Liu *et al.*, 2018). In non-emasculated *Sldad1* ovaries at 0 DAA and 4 DAA, *SlARF5* and *SlARF7* were down-regulated (Fig. 7D). In contrast, *SlARF6A* (*Solyc12g006340*), *SlARF6B-1* (*Solyc07g043620*), *SlARF6B-2* (*Solyc07g043620*), and *SlARF8A/SlARF8B*, which are predominantly found in the pericarp, septum, and placenta, showed no significant differences between WT and *Sldad1* ovaries. These results indicated that GA as well as auxin and potentially other phytohormones, are involved in the regulation of tomato fruit set.

## Discussion

### JA synthesized in filaments probably accumulates in ovules to suppress fruit set

Our results identified *SlDAD1* (*Solyc10g038170*) as the gene responsible for the *Sldad1* mutant in tomato. The SlDAD1 protein sequence shares high homology with Arabidopsis DAD1, which is a plastid-located enzyme involved in the initial steps of JA biosynthesis (Ishiguro *et al.*, 2001). Notably, *SlDAD1*, which catalyses the conversion of phospholipid to α-LA, was predominantly transcribed in WT filaments before anthesis. We observed peak *SlDAD1* expression levels at −2 DAA, which was accompanied by JA accumulation in ovules (Figs 3, 4C). Interestingly, JA and its bioactive form JA-Ile accumulated not only in the filament but also in the ovary before anthesis (Fig. 3B, C). The −2 DAA stage is critical for coordinating anther dehiscence, ovule maturation, and petal opening (Ishiguro *et al.*, 2001; Schubert *et al.*, 2019). In contrast, in the *Sldad1* mutant, JA and JA-Ile were relatively undetectable in both the filament and ovary across developmental stages. Similarly, *AtDAD1* has been shown to be specifically expressed in the filament just before anthesis, and RNA-seq data in Arabidopsis confirms its filament-specific expression (Ishiguro *et al.*, 2001; Klepikova *et al.*, 2016). Our findings are consistent with a previous study showing JA accumulation in the ovary/ovule of the *jai1-1* mutant at −2 DAA (Schubert *et al.*, 2019). Given that JA and JA-Ile concentrations in WT ovaries decreased in the approach to anthesis alongside reduced *SlDAD1* transcription, it suggests that the role of JA in tomato fruit set is critical before rather than after anthesis. This hypothesis is supported by studies on the tomato MADS-box mutant *Sltap3*, where stamen with carpeloid characteristics exhibit reduced JA concentration compared to unfertilized WT ovaries (Okabe *et al.*, 2019; Kusano *et al.*, 2022). These results suggest that JA synthesized in the filaments might be transported to the ovaries, where it suppresses fruit set. This is further supported by our DESI–MSI and immunocytological analyses, which showed that JA and JA-Ile predominantly accumulated in WT filaments and ovules, peaking at −2 DAA, with levels significantly decreasing by anthesis (Fig. 4). Our mass-imaging data demonstrated the utility of detailed JA imaging in tomato tissues, especially during the early stages before anthesis, highlighting DESI–MSI as a powerful tool for visualizing JA. Transcript profiling of JA-related genes revealed that both biosynthesis and signaling genes tended to be up-regulated in WT filaments at −2 DAA, while signaling genes alone were primarily up-regulated in ovaries at −2 DAA (Fig. 7B). Additionally, the expression patterns of tomato JA transporter genes, including the *JAT* (Li *et al.*, 2020), *JASSY* (Guan *et al.*, 2019), and *GTR/NPF2.10* (Saito *et al.*, 2015) orthologs of Arabidopsis JA transporters, were up-regulated in WT filaments at anthesis but not pre-anthesis. In contrast, these genes were down-regulated in the *Sldad1* mutant at all stages (Fig. 7B). Although direct movement has yet to be observed, our findings suggest that JA synthesized in the filaments is probably transported to the ovules before anthesis, thereby inhibiting fruit set in tomato. Our data indicate that the role of JA in tomato fruit initiation might involve the regulation of ovule development before anthesis, similar to the importance of JA in Arabidopsis male fertility (Jewell and Browse, 2016). For instance, the JA biosynthetic enzyme ALLENE OXIDE CYCLASE (AOC) is specifically localized within ovules (Hause *et al.*, 2000). While we cannot rule out the possibility that JA biosynthesis might also occur in the ovules themselves, our findings highlight the essential role of filament-derived JA in the early stages of tomato fruit development.

### JA perception, rather than biosynthesis, is crucial for female fertility

JA has been shown to play a key role in late ovule development, as demonstrated by analysis of the *jai1-1* mutant that is deficient in JA perception (Schubert *et al.*, 2019). As well as female sterility, the *jai1-1* mutant exhibits male sterility, characterized by partially reduced pollen viability, impaired germination, and premature anther dehiscence (Dobritzsch *et al.*, 2015; Schubert *et al.*, 2019). When crossed with either WT or *jai1-1* pollen grains, *jai1-1* fails to produce seeds (Li *et al.*, 2004). Although ovule initiation appears normal, arrested embryo sac development can impair female fertility (Schubert *et al.*, 2019). In *jai1-1* and *Slmyb21* mutants, where JA/JA-Ile levels are reduced in the ovaries, vacuolated cells are formed around the embryo sac, accompanied by callose deposition and programmed cell death (Schubert *et al.*, 2019). *SlMYB21* expression is markedly down-regulated in *jai1-1* mutants, and ovules in both the *jai1-1* and *Slmyb21* mutants display increased callose deposition and cell vacuolation within the embryo sac; this leads to the premature breakdown of the nucellus before fertilization, causing female sterility (Schubert *et al.*, 2019). Notably, *Slmyb21* mutants do not exhibit early stamen senescence or significant loss of pollen viability (Li *et al.*, 2004; Dobritzsch *et al.*, 2015; Schubert *et al.*, 2019). Under normal conditions, *Slmyb21* plants develop fruits similar to the WT, with seedless fruit formation, but produce some seeds when hand-pollinated (Schubert *et al.*, 2019). Overexpression of *AtMYB24-SRDX* in tomato results in aborted flower opening, similar to *jai1-1*, as well as defective pollen grains and pistils, causing both male and female sterility (Niwa *et al.*, 2018). In contrast, we observed no significant differences in anther structure or pollen germination rates the WT and *Sldad1* mutant (Supplementary Fig. S2A–E). Additionally, *Sldad1* produced seeds upon self-pollination (Fig. 1D), indicating that *Sldad1* pollen remains fertile. Consistent with these findings, JA was specifically detected around the embryo sac in WT ovules at −2 DAA but not in *Sldad1* ovules (Fig. 4C). Together, these results suggest that the effects of ovule development in *Sldad1* mutants are less severe than those in *jai1-1* and *Slmyb21*, indicating that JA perception, rather than JA biosynthesis, plays a more critical role in maintaining female fertility in tomato.

### *Sldad1*-induced fruit set is primarily driven by cell expansion due to increased GA levels

We observed that ovary growth without manual pollination in *Sldad1* was probably the result of a significant increase in cell size within the ovary wall (Fig. 5). Concentrations of bioactive GAs (GA_1_ and GA_4_) were markedly increased in non-emasculated *Sldad1* ovaries at 4 DAA; GA_1_ levels were comparable to those observed in pollinated WT and *Sldad1* ovaries (Fig. 6A). This increase was consistent with elevated mRNA levels of GA biosynthesis genes (*SlGA20ox1* and *SlGA20ox3*) and reduced expression of GA inactivation genes (*SlGA2ox4* and *SlGA2ox5*) (Fig. 7C). Furthermore, application of the GA biosynthesis inhibitor PAC inhibited most *Sldad1*-induced fruit set (Fig. 6B), suggesting that JA acts, at least in part, through GA in the regulation of tomato ovary development. Consistent with this, a study of the *jai1-1* mutant has shown that JA reduces GA accumulation (Schubert *et al.*, 2019). Phenotypes such as the increased height of *Sldad1* plants compared with the WT and the persistence of styles and petals, similar to those of the *procera* mutant, support the role of elevated GA levels in driving these traits (Carrera *et al.*, 2012; Shinozaki *et al.*, 2018). These findings suggest that fruit development in the *Sldad1* mutant is at least partially mediated by activation of the GA pathway.

The increased number of cell layers before anthesis in *Sldad1* ovaries (Fig. 5E), accompanied by high IAA concentrations (Fig. 6C) and the up-regulation of genes related to cell division (Supplementary Fig. S14B), suggests that auxin might facilitate cell division and promote early ovary growth prior to anthesis. Mutations in *SlARF5* and *SlARF7* are known to result in parthenocarpy (de Jong *et al.*, 2009; Liu *et al.*, 2018). Expression levels of *SlARF5* and *SlARF7* were down-regulated in non-emasculated *Sldad1* ovaries at 4 DAA (Fig. 7D). In Arabidopsis, ARF6 and ARF8 enhance the expression of JA biosynthesis genes in the filament (Nagpal *et al.*, 2005), and the *arf6/arf8* mutant displays delayed flower-bud opening, a phenotype similar to that of the *Atdad1* mutant, which is characterized by the down-regulation of *AtDAD1* and other JA biosynthesis genes, suggesting that ARF6 and ARF8 are required for the activation of *DAD1* expression (Ishiguro *et al.*, 2001; Tabata *et al.*, 2010). In tomato, suppression of ARF6 and ARF8 by microRNA167 leads to abnormal floral development and female sterility (Liu *et al.*, 2014). ARF8 is considered a negative regulator of fruit initiation, and hence its suppression induces parthenocarpy in both Arabidopsis and tomato (Goetz *et al.*, 2006, 2007). In our ovary transcriptome profile, no significant expression changes were observed for *SlARF6A/SlARF6B* and *SlARF8A/SlARF8B*, although recent findings indicate reduced JA concentration in the ovaries of the parthenocarpic *Slarf8a/8b* mutant (Israeli *et al.*, 2023). The changes in size in *Sldad1* ovaries occurred before anthesis, around −5 DAA (Fig. 5A), suggesting that JA was regulated by auxin signaling through *SlARF6* and *SlARF8* prior to anthesis. Auxin increases the mRNA levels of the copalyl diphosphate synthase genes *SlCPS1* and *SlGA20ox1* (Serrani *et al.*, 2008, 2010; Mariotti *et al.*, 2011). Consistent with this, *SlGA20ox1* was upregulated in non-emasculated *Sldad1* ovaries at 4 DAA (Fig. 7C). Given the constitutively high concentrations of IAA before anthesis, the absence of JA might also influence GA levels through auxin during early ovary development. Further studies are required to clarify the regulatory mechanism linking JA signaling downstream of *SlDAD1* to auxin and GA signaling.

### JA potentially influences the metabolism of CKs, ABA, and ethylene during early ovary development

Fruit set in tomato is regulated not only by GA and auxin but also by other phytohormones, such as CKs, ABA, and ethylene (ET) (Shinozaki *et al.*, 2015). CKs play a critical role in inducing seedless fruits through cell division (Matsuo *et al.*, 2012). In non-emasculated *Sldad1* ovaries, the concentrations of active CKs (*t*ZR, *t*Z, iPR, and iP) were either stable or increased during early ovary development (Supplementary Fig. S11A). ABA is also considered important for tomato fruit set (Nitsch *et al.*, 2009; Shinozaki *et al.*, 2015), although its exact role remains unclear. ABA levels are typically high before anthesis and decrease following pollination, suggesting that it might help suppress development before fruit set (Vriezen *et al.*, 2008; Shinozaki *et al.*, 2015). We found that ABA concentrations were high in non-emasculated WT and *Sldad1* ovaries from −2 DAA to 0 DAA, and then decreased in pollinated WT and *Sldad1* ovaries at 2 DAA and 4 DAA (Supplementary Fig. S11B). The concentrations were higher in non-emasculated *Sldad1* ovaries compared with those of pollinated ovaries, suggesting that JA might influence ABA metabolism during early ovary development. ET also plays a role in tomato fruit set (Shinozaki *et al.*, 2015). In Arabidopsis, it is involved in anther development through the regulation of JA. We observed that the *Sldad1* mutant and genome-edited lines (#*42*-*1*_*1* and #*42*-*1*_*2*) often displayed anther browning and failure in enlarging some ovaries in inflorescences and inducing abscission of petals and styles (Supplementary Fig. S8C, E, F). This indicates that ET might also contribute to fruit set by preventing immature ovule development and ensuring the proper timing of pre-fertilization events in tomato, potentially mediated by JA. Taken together, the pollination-dependent fruit set in *Sldad1* occurred via a mechanism similar to that in WT plants, while seedless fruit production in *Sldad1* is probably influenced by CKs, ABA, and ET.

### Is the *Sldad1* mutant parthenocarpic or seedless?

**Fig. 8.**
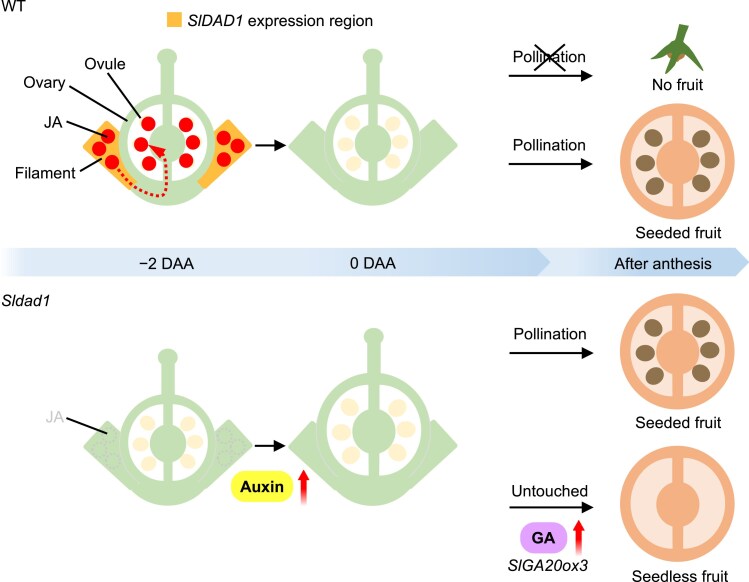
A working model for JA-mediated regulation of tomato fruit development. In the wild type (WT), the jasmonic acid (JA) biosynthesis gene *SlDAD1* is specifically expressed in the filaments before anthesis, particularly at −2 days after anthesis (DAA). During this period, JA levels (red) transiently increase in the filaments and ovules, thereby suppressing ovary enlargement prior to anthesis. Consequently, in the absence of pollination the ovaries fail to develop into fruits. The signal, which is probably emitted through the ovules (dotted arrow), is not clear. In contrast, the *Sldad1* mutant exhibits markedly low JA levels in both the filaments and ovules before anthesis. These reduced levels probably promote ovary enlargement through enhanced cell division and expansion, mediated by phytohormones such as auxin and gibberellin (GA) via increased expression of SlGA20-oxidases, leading to seedless fruit set in untouched flowers (i.e. without any manual pollination or emasculation). However, the molecular mechanism of how the *Sldad1* mutation induces seedless fruits is still unclear.

In this study, we identified a novel gene in tomato, *SlDAD1*, that is involved in the production of seedless fruits. The fruit phenotype of the *Sldad1* mutant raises the question of whether it should be classified as parthenocarpy or seedless. We observed stigma exsertion in all flowers of non-emasculated *Sldad1* plants at 0 DAA (Supplementary Fig. S3B) when anther dehiscence occurred in the mutant (Supplementary Fig. S2A), indicating that self-pollination is physically unlikely to occur. To further support this, the presence of pollen was not observed in untouched flowers after anthesis (Supplementary Fig. S1C); this suggests that pollination did not seem to occur in the mutant. These observations support fruit set in the mutant being initiated independently of pollination. Hand-pollinated and emasculated flowers in the mutant showed a markedly higher rate of fruit set compared with those that were only emasculated without hand pollination, where the rate of fruit set was ∼2%. Furthermore, *Sldad1* could set fruits and produce seeds when pollinated, regardless of emasculation (Fig. 1D; Supplementary Fig. S1A); this implies that fertilization can occur, although it might sometimes be incomplete following emasculation. When the mutant was pollinated without emasculation, all fruits produced seeds (Fig. 1D). Therefore, the formation of seedless fruits suggests that pollination did not occur. In addition, the *Sldad1* allele in the ‘Ueleie 106 WP’ background exhibited a high fruit set after emasculation (Supplementary Fig. S6E), suggesting that fruit set might occur even when the anther is removed. However, the possibility that pollination occurred without any manual pollination or emasculation (i.e. untouched) cannot be completely ruled out. The results of our emasculation tests raise two possibilities. One is that wound-induced JA production triggered by emasculation might suppress ovary development in the *Sldad1* mutant. Supporting this possibility, *SlDAD1* expression was induced after emasculation in the *Sldad1* mutant (Supplementary Fig. S3F, G). The second possibility is that signals originating from stamens might inhibit ovary growth in the mutant. Mutations in *HYDRA*/*SPOROCYTELESS*-like that result in seedless fruit suggest that signals generated during male gametogenesis might inhibit tomato ovary development (Hao *et al.*, 2017), although the underlying molecular mechanism remains unclear. *SlDAD1* was specifically transcribed in the filament before anthesis, and the JA concentration was correlated with its transcript levels in the filament (Fig. 3). JA also accumulated in the ovary before anthesis. Phytohormone profiling and transcriptome analysis indicated that *SlGA20ox3*-mediated GA metabolism was enhanced by the absence of JA accumulation (Fig. 7). These findings suggest that spatial and temporal control of *SlDAD1* transcripts and JA/JA-Ile levels might regulate fruit set by coordinating flower development. We propose that JA serves to delay ovule maturation before anthesis, suppresses GA, and supports phytohormone crosstalk (Fig. 8). Previous studies have indicated a relationship between embryonic defects and seedless fruit development, such as in the *Slagl6* and *Slhb15a* mutants (Klap *et al.*, 2017; Clepet *et al.*, 2021; Gupta *et al.*, 2021), and some of the results of this study are consistent with those findings. However, the precise mechanism by which JA accumulates in the innermost ovule integument before anthesis to regulate fruit set remains unknown. Further research is needed to clarify the potential role of tissue-specific localization of phytohormones in controlling tomato fruit set.

## Supplementary Material

eraf349_Supplementary_Data

## Data Availability

The RNA-seq raw data are available in the Sequence Read Archive (SRA) database of the DNA Data Bank of Japan (DDBJ) at (https://www.ddbj.nig.ac.jp/index.html) under bioproject PRJDB20494. The other data supporting the findings of this study are available from the corresponding author upon request.

## Supplementary data

The following supplementary data are available at *JXB* online.

Fig. S1. Phenotypes of the WT and *Sldad1* mutant.

Fig. S2. Male reproductive development and ovule morphology of the WT and *Sldad1* mutant.

Fig. S3. Flowering stages, expression patterns of *SlDAD1*, and JA concentrations in different floral organs.

Fig. S4. Phenotype segregation indicating that the *Sldad1* mutation is monogenic recessive.

Fig. S5. Genotype mapping of F_2_ seedless plants crossed between *Sldad1* and ‘Resina’.

Fig. S6. Candidate SNP mutations identified by next-generation sequencing.

Fig. S7. Amino acid sequence alignment of Arabidopsis and tomato DAD1 proteins.

Fig. S8. S*lDAD1* mutations generated by CRISPR/Cas9.

Fig. S9. Endogenous levels of JA in ovaries.

Fig. S10. Endogenous concentrations of GAs in WT and *Sldad1* ovaries.

Fig. S11. Endogenous concentrations of CKs and ABA in WT and *Sldad1* ovaries.

Fig. S12. Validation of expression of JA biosynthesis genes by qRT-PCR.

Fig. S13. Venn diagrams of up- and down-regulated DEGs in *Sldad1* ovaries at different time-points.

Fig. S14. GO enrichment analysis of non-emasculated WT and *Sldad1* ovaries from −2 DAA and 0 DAA.

Table S1. Sequences of primers used in this study.

Table S2. Up-regulated DEGs between *Sldad1* and the WT.

Table S3. Down-regulated DEGs between *Sldad1* and the WT.
