# Supplementary material for: Spatiotemporal localization of jasmonate in the regulation of fruit set in tomato
Source: J Exp Bot. 2025 Aug 1;76(21):6527–47. doi: 10.1093/jxb/eraf349 (PMC12646150; doi:10.1093/jxb/eraf349)
Supplement: eraf349_Supplementary_Data [file eraf349_supplementary_data.zip › NEW_Suppl_Figs_S1-14.pdf]

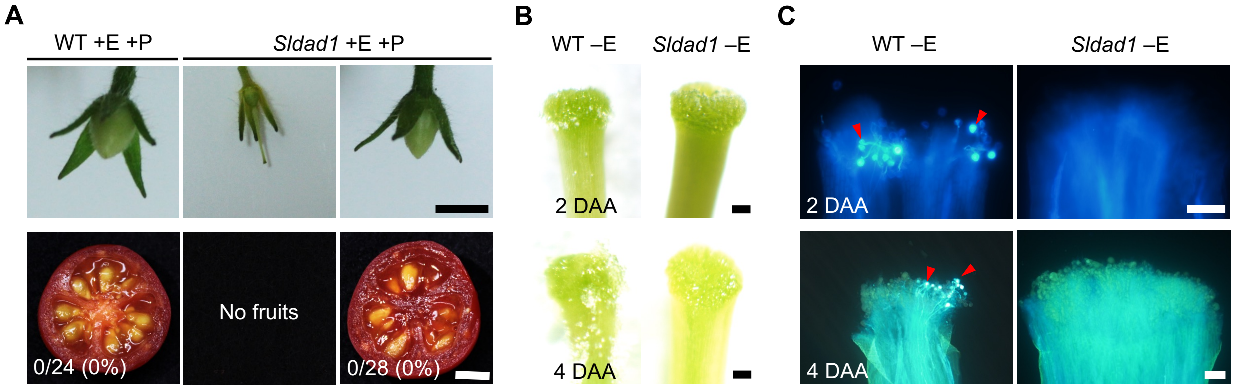

D

	WT –E	*Sldad1* –E	*Sldad1* –E +P
Single fruit weight (g)	3.09 ± 0.19 ns	2.43 ± 0.30 ns	3.73 ± 0.46 ns
Fruit length (mm)	20.42 ± 0.39 a	16.49 ± 0.63 b	17.69 ± 0.46 b
Fruit diameter (mm)	18.96 ± 0.42 ns	18.01 ± 1.06 ns	17.85 ± 0.58 ns
Columella weight (g)	0.42 ± 0.03 ns	0.48 ± 0.09 ns	0.52 ± 0.06 ns
Pericarp weight (g)	1.99 ± 0.11 ns	1.59 ± 0.29 ns	1.96 ± 0.15 ns
Locular gel weight (g)	0.20 ± 0.01 b	0.33 ± 0.05 a	0.26 ± 0.03 ab
Seed number per fruit (n)	14.59 ± 1.70 a	0 b	11.80 ± 2.15 a
100-seed weight (g)	0.27 ± 0.01 b	–	0.30 ± 0.00 a
Locular number (n)	3.06 ± 0.07 a	3.40 ± 0.32 a	2.80 ± 0.16 b

Fig. S1. Phenotypes of the WT and *Sldad1* mutant. (A) Phenotype of pollinated WT and *Sldad1* mutant at 4 DAA (upper) and the red stage (lower) after emasculation. Numbers indicate the number of seedless fruits and seedless fruit ratio are shown in parentheses. Scale bar is 5 mm. (B, C) The presence of pollen on WT and *Sldad1* stigma at 2 (upper) and 4 DAA (lower) using a stereomicroscope (B) and aniline blue staining observed under fluorescence microscopy (C). Red arrowheads indicate pollen grains on the stigma (C). Scale bar is 100 µm. (D) Comparison of reproductive phenotypes between non-emasculated WT and *Sldad1* fruits in the red stage. Data are mean (±SE), WT: *n*=25; *Sldad1*: *n*=15. All data were obtained from five fruits per plant and a minimum of three plants. Mature dried seeds of WT and *Sldad1* mutant were used to calculate the 100-seed weight. Different letters indicate significant differences (**P*<0.05; Tukey–Kramer test). ns, not significant; DAA, days after anthesis; –E, non-emasculated; +E, emasculated; +P, pollinated.

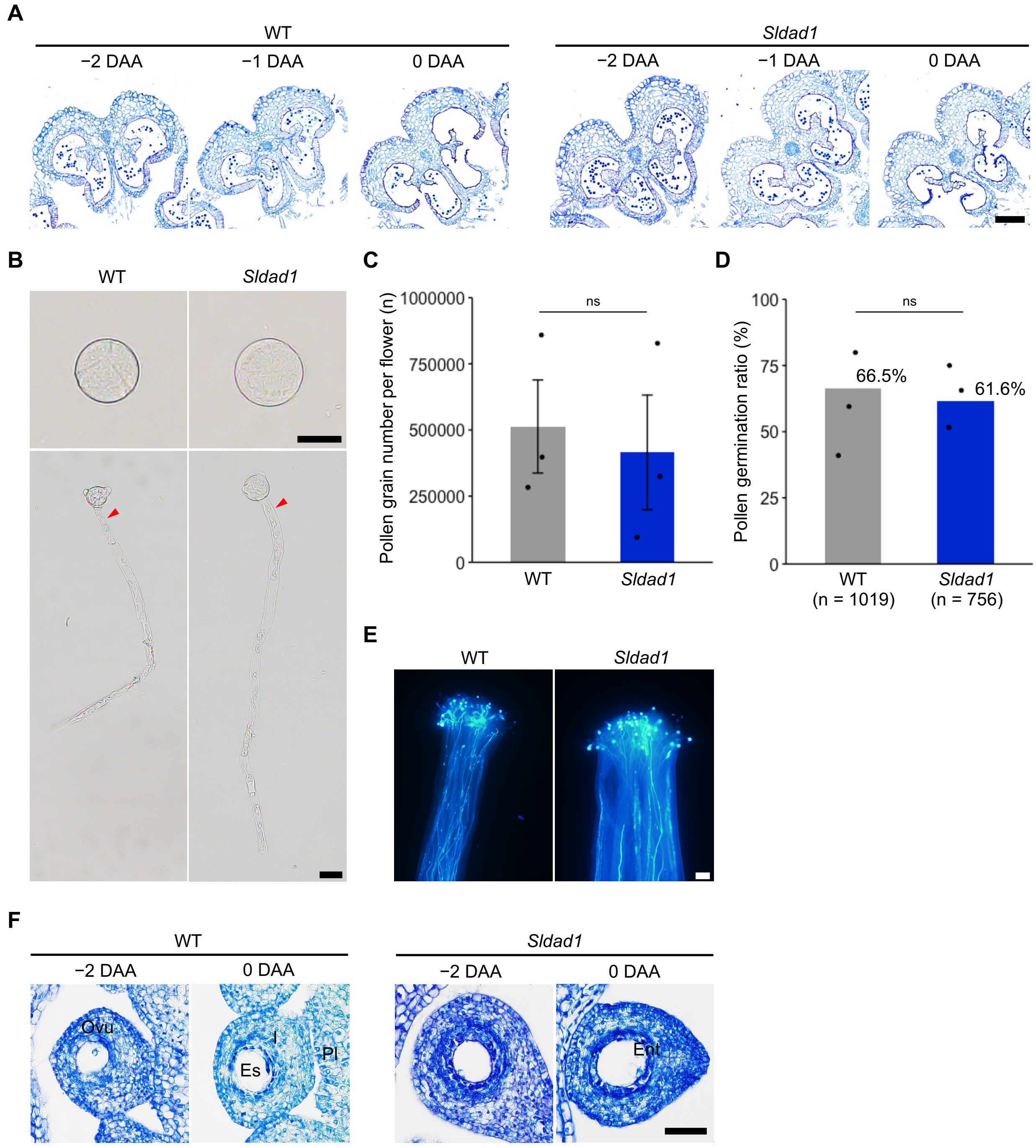

Fig. S2. Male reproductive development and ovule morphology of the WT and *Sldad1* mutant. (A) Transverse sections of WT and *Sldad1* anthers at −2, −1 and 0 DAA. Scale bar is 200 μm. (B) Pollen grain and pollen germination of WT and *Sldad1* in the germination media. Red arrowheads show germinated pollen tube. Scale bar is 20 μm. (C) Pollen grain number and (D) pollen germination ratio of WT and *Sldad1* in the germination media. Data are mean (±SE), n=3. n.s. indicate no significant differences (*P<0.05; Welch t-test). (E) Pollen germination on stigma for WT and *Sldad1* mutant two days after pollination. Scale bar is 100 μm. (F) Morphology of WT and *Sldad1* ovules at −2 and 0 DAA. Scale bar is 50 μm. Ovu, ovule; Es, embryo sac; I, integument; Pl, placenta; Ent, endothelium; DAA, days after anthesis.

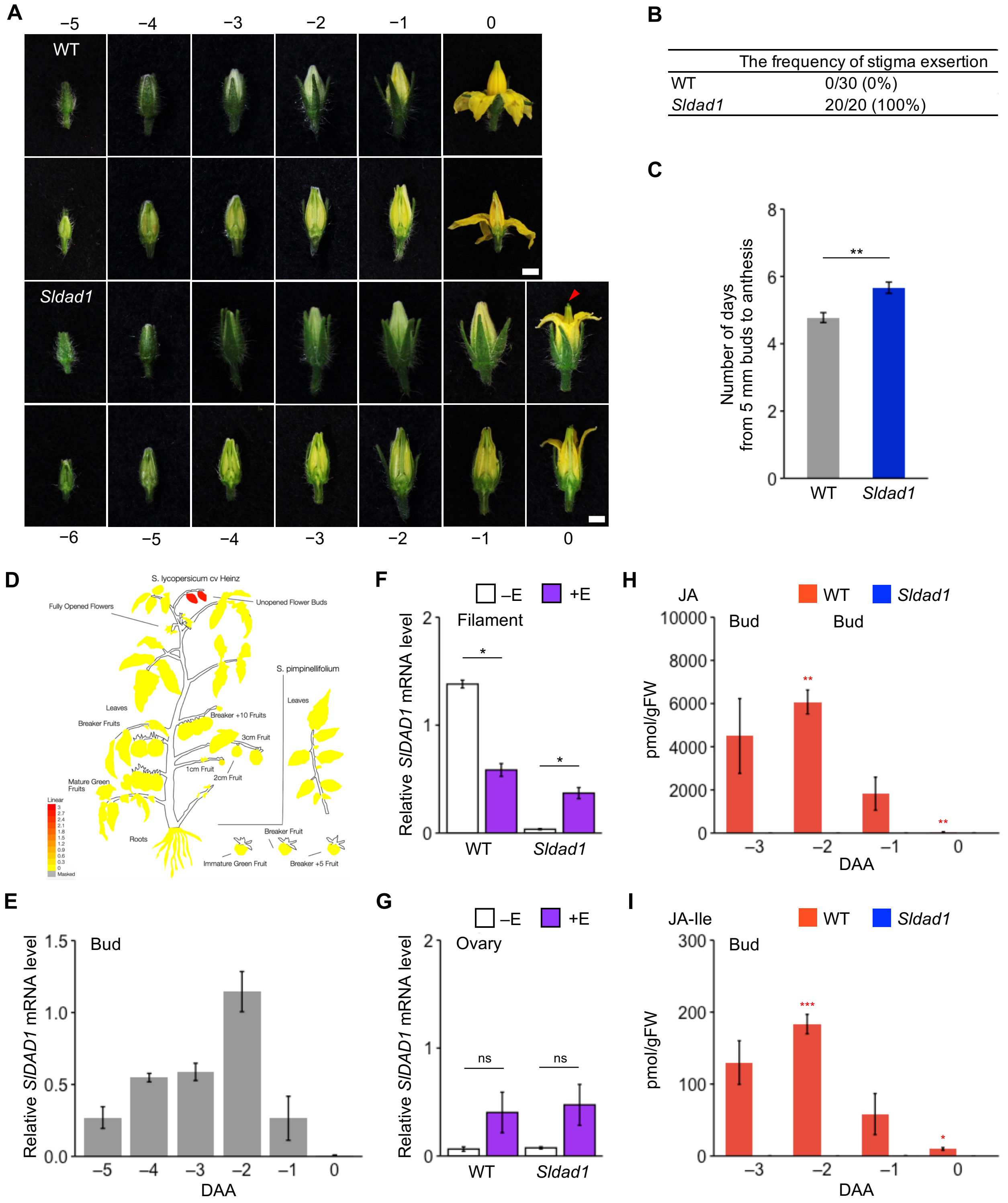

Fig. S3. Flowering stages, expression patterns of *SlDAD1*, and JA concentrations in different floral organs. (A) Flowers of WT and *Sldad1* mutant. Flowering defined for this study. Numbers represent days after anthesis. Red arrowhead indicates stigma exsertion. *Sldad1* petals did not fully open. Scale bar is 2 mm. (B) Quantification of the frequency of stigma exsertion. The stigma exsertion was observed from 0 DAA. (C) Days from 5 mm buds to anthesis. Data are mean (±SE), *n*=9. Asterisks indicate significant differences (***P*<0.01; Welch *t*-test). (D) Predicted expression level of *SlDAD1* in Tomato eFP browser (http://bar.utoronto.ca/eplant_tomato/). Yellow color shows minimum and red color shows maximum expression. (E) *SlDAD1* mRNA level in WT buds by qRT-PCR. *SAND* was used for internal control. Data are mean (±SE), *n*=4. (F, G) *SlDAD1* mRNA level in the filament (F) and ovary (G) 1 hour after anther removal (emasculation). *SAND* was used for internal control. Data are mean (±SE), *n*=3. (H, I) Concentrations of (H) JA and (I) JA-Ile concentration in the buds from −3 to 0 DAA. Data are mean (±SE), *n*=4. Asterisks indicate significant differences from WT (**P*<0.05, ***P*<0.01, ****P*<0.001; Welch *t*-test) (F–I). JA, jasmonic acid; JA-Ile, JA-isoleucine; ns, not significant; DAA, days after anthesis; –E, non-emasculated; +E, emasculated.

	‘Micro-Tom’ WT × *W2939* (BC_1F_2)
Number of parthenocarpy plants	48
Number of non-parthenocarpy plants	156
chi-square text *p*-value (expected 1:3)	0.63

Fig. S4. Phenotype segregation indicating that the *Sldad1* mutation is monogenic recessive. Phenotype segregation of the F_2 plants was shown.

Marker	Chromosome number	'Micro-Tom'	'Regina'	Genotypes of each F_2 smapping population												Frequency of 'M' (%)
				1	2	3	4	5	6	7	8	9	10	11	12	
6172_1047	1S	M	R	R	M	M	M	R	R	M	H	M	H	H	M	50.0
273_877	1L	M	R	R	H	H	M	R	R	H	H	H	H	H	H	8.3
7111_1701	2S	M	R	R	R	R	R	R	R	R	H	R	M	M	M	25.0
9673_646	2L	M	R	R	R	H	R	R	R	H	R	R	H	H	M	8.3
3351_2089	3S	M	R	H	H	R	H	H	M	H	R	M	R	H	M	25.0
43_1626	3L	M	R	H	R	H	H	M	M	H	H	H	R	M	M	33.3
6643_455	4S	M	R	H	H	M	H	H	M	R	R	M	H	H	M	33.3
837_663	4L	M	R	H	H	H	H	H	H	M	H	R	H	H	H	8.3
7138_174	5S	M	R	H	M	M	M	M	R	H	H	R	M	H	M	50.0
9768_1494	5L	M	R	R	M	M	M	M	H	H	H	H	M	H	M	50.0
7100_120	6S	M	R	H	H	H	H	H	R	R	H	M	H	R	H	8.3
6651_568	6L	M	R	H	M	R	R	R	R	M	H	H	M	R	R	25.0
9024_376	7S	M	R	H	R	H	H	R	M	H	M	R	H	H	H	16.7
2971_740	7L	M	R	H	M	H	H	H	M	M	H	M	H	H	R	33.3
10489_192	8S	M	R	R	H	H	H	R	H	H	M	H	M	H	H	16.7
3538_583	8L	M	R	M	H	M	M	R	M	H	H	H	M	H	R	41.7
16586_441	9S	M	R	H	M	H	H	M	M	H	H	H	H	M	H	33.3
2910_740	9L	M	R	H	R	H	H	H	M	H	H	M	H	M	M	33.3
2120_1008	10S	M	R	M	H	M	M	M	M	M	M	M	M	H	M	83.3
13536_438	10L	M	R	H	M	M	H	M	M	M	H	H	M	M	M	66.7
2874_1225	11S	M	R	H	M	M	M	M	M	H	H	M	M	H	M	66.7
18095_307	11L	M	R	R	M	H	H	H	H	H	M	H	M	H	M	33.3
9542_306	12S	M	R	R	M	M	M	R	H	M	H	M	M	M	R	58.3
2559_638	12L	M	R	H	H	R	H	H	M	H	M	H	H	H	M	25.0

Fig. S5. Genotype mapping of F_2 seedless plants crossed between *Sldad1* and 'Resina'. Twelve F_2 seedless plants crossed between *Sldad1* and 'Resina' were used for genotype mapping, and ten of them showed homozygous 'Micro-Tom' genotype on the short arm of chromosome 10. DNA marker information is available in tomato marker database (http://marker.kazusa.or.jp/tomato/). M, homozygous genotype of 'Micro-Tom'; R, homozygous genotype of 'Resina'; H, heterozygous genotype; S, short arm; L, long arm.

A

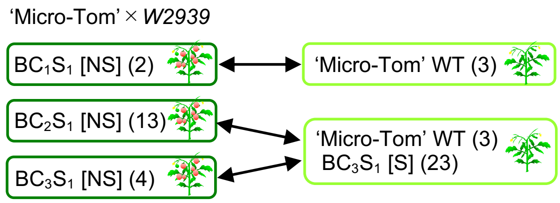

B

Generation	Reference	Chr Position	WT genotype	*W2939* genotype	DP	GQ score	Gene ID	Annotation	Mutation position	Effect of mutation
BC_1S_1 [P]	SL2.50	20398477	C	T	40	99	*Solyc10g038170*	DEFECTIVE ANTHER DEHISCENCE 1; Lipase class 3	exon	Missense
		20398514	C	T	44	99	*Solyc10g038170*	DEFECTIVE ANTHER DEHISCENCE 1; Lipase class 3	exon	Missense
		38090627	G	A	28	84	*Solyc10g046900*	Unknown protein	intron	Missense
BC_2S_1 [P]	SL3.0	11257245	C	A	41	99	*Solyc10g019033*	Unknown protein	exon	Missense
		20373356	C	T	50	99	*Solyc10g038170*	DEFECTIVE ANTHER DEHISCENCE 1; Lipase class 3	exon	Missense
		20373393	C	T	51	99	*Solyc10g038170*	DEFECTIVE ANTHER DEHISCENCE 1; Lipase class 3	exon	Missense
BC_3S_1 [P]	SL3.0	3695385	G	C	65	99	*Solyc10g009590*	TRICHOME BIREFRINGENCE-LIKE 25	exon	Missense
		3823305	G	A	8	24	*Solyc10g009640*	AtGH3.11; Auxin-responsive GH3 family protein	exon	Missense
		11257245	C	A	60	99	*Solyc10g019033*	Unknown protein	exon	Missense
		20373356	C	T	67	99	*Solyc10g038170*	DEFECTIVE ANTHER DEHISCENCE 1; Lipase class 3	exon	Missense
		20373393	C	T	66	99	*Solyc10g038170*	DEFECTIVE ANTHER DEHISCENCE 1; Lipase class 3	exon	Missense

C

	Non-seed plants	Seeded plants
Homozygous (WT/WT)	–	13
Hetrozygous (WT/*mt*)	–	19
Homozygous (*mt*/*mt*)	21	–

D

Gene ID	RPKM average	
	[NS]	[S]
Solyc10g019033	0.00	0.00
Solyc10g038170	0.02	0.03

E

Line	Seedless genotype	Number of emasculation	Number of fruit set	Fruit set ratio (%)
'Ueleie 106 WP' WT	+/+	80	0	0
'Ueleie 106 WP' × *W2939* F_3 Non-heat resistance	+/+ or +/m	80	11	13.8
'Ueleie 106 WP' × *W2939* F_3 Heat resistance	m/m	80	46	57.5

F

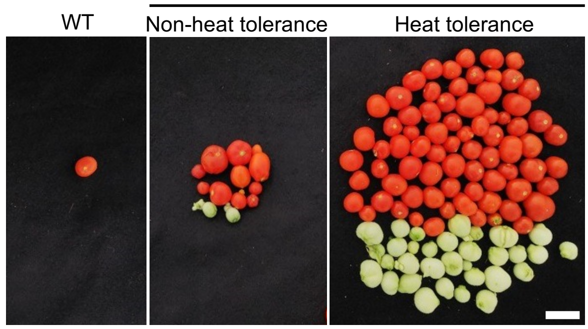

G

Line	Seedless genotype	Number of emasculation	Number of fruit set	Fruit set ratio (%)
'Micro-Tom' WT	+/+	77	3	3.9
'Micro-Tom' × *W2939* BC_2S_2 [P]	m/m	63	12	19.0

H

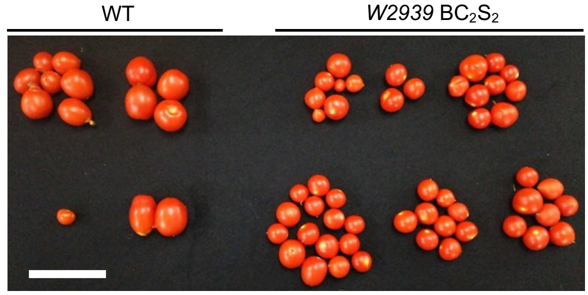

Fig. S6. Candidate SNP mutations identified by next-generation sequencing. (A) Overview of the approach used for causative gene identification using whole genome sequencing. The plant number is shown in parenthesis. Of the SNPs detected, mutations common to the above three comparisons were judged as candidate genes. (B) Mutations with non-synonymous substitution of amino acids (SNP-index = 1, DP≥5, QO>20) were selected. A candidate gene common to all is highlighted. Chr position, DP, and GQ score indicate the physical position of mutation on the tomato reference genome (SL2.50 or SL3.0), the read depth, and the quality of the assigned genotype, respectively. (C) Allelism test with 'Micro-Tom' WT × *W2939* BC_3S_1 plants. (D) RPKM of ovaries at 2 DAA in two candidate genes. n=3. (E–H) Yield of F_3 plants crossed between *Sldad1* in 'Micro-Tom' and WT in 'Ueleie 106 WP' (E, F) and between *Sldad1* in 'Micro-Tom' and WT in 'Micro-Tom' (G, H) under greenhouse conditions. (E, G) Seedless fruit ratio of WT and F_3 plants (E) and BC_2S_2 plants (G). Four plants were used and twenty flowers were emasculated per plant (E) and four or six plants were used and flowers were emasculated (G) at two days before anthesis. (F, H) Representative pictures of fruit yield per plant in WT and *Sldad1*. Fruits from the third flower cluster were harvested. Scale bars are 5 cm. Plants were grown under 25–45°C day/17–27°C night in summer 2018 (E, F) and 28–45°C day/20–27°C night in summer 2017 (G, H). RPKM, reads per kilobase of exon per million mapped reads; DAA, days after anthesis; NS, non-seed; S, seeded.

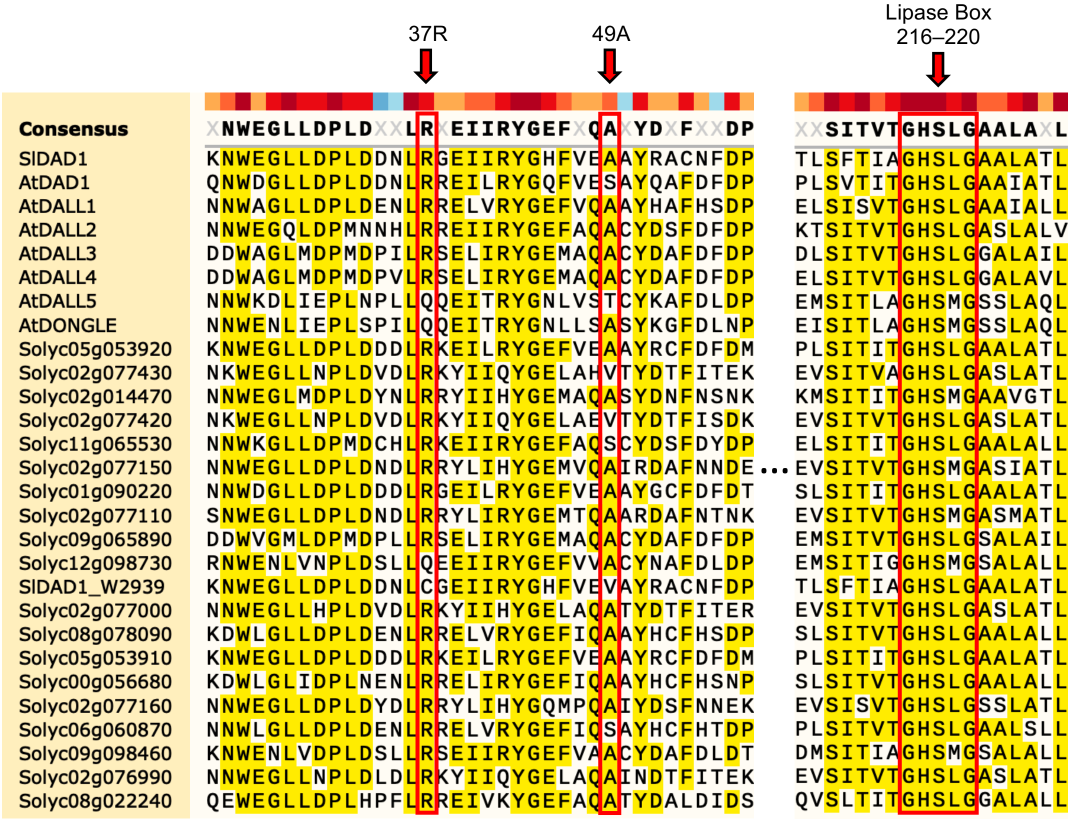

Fig. S7. Amino acid sequences alignment of Arabidopsis and tomato DAD1 proteins. *Sldad1* mutation sites are conserved among phospholipase A1. R (arginine) was conserved in SlDAD1 and AtDAD1. Red boxes indicate mutation sites.

A

gRNA1

Line	Sequence (5'-3')	Δbp	Genotype
WT	CCACTAGATGATAATTT−ACGTGG		
#3-1	CCACTAGATGATAATTTTACGTGG	+1	Homo
#9-1	CCACTAGATGATAATTT−ACGTGG<br>CCACTAGATGATAATTTTACGTGG	WT<br>+1	Hetero
#9-3	−	−	Chimera
#9-4	−	−	Chimera
#10-1	CCACTAGATGATAATTT−ACGTGG<br>CCACTAGATGATAATTTTACGTGG	WT<br>+1	Hetero
#25-1	CCACTAGATGATAATTTTACGTGG<br>CCACTAGATGATAAT−−−ACGTGG	+1<br>−2	Biallelic
#37-1	CCACTAGATGATAATTT−ACGTGG<br>CCACTAGATGATAATTTTACGTGG	WT<br>+1	Hetero
#42-1	CCACTAGATGATAATTTTACGTGG<br>CCACTAGATGATAAT−−−ACGTGG	+1<br>−2	Biallelic

gRNA2

Line	Sequence (5'-3')	Δbp	Genotype
WT	TATTTGCACATTGCGTATGAAGG		
#3-1	TATTTGCACATTGCGTATGAAGG	WT	WT
#9-1	TATTTGCACATTGCGTATGAAGG	WT	WT
#9-3	TATTTGCACATTGCGTATGAAGG	WT	WT
#9-4	TATTTGCACATTGCGTATGAAGG	WT	WT
#10-1	TATTTGCACATTGCGTATGAAGG	WT	WT
#25-1	TATTTGCACATTGCGTATGAAGG	WT	WT
#37-1	TATTTGCACATTGCGTATGAAGG	WT	WT
#42-1	TATTTGCACATTGCGTATGAAGG<br>TATTTGCACA−−−−−−−TGAAGG	WT<br>−7	Hetero

B

Line	Sequence (5'-3')	Mutation	Length
WT	LDDNLRGEIIRYGHFVEAAY	−	376 a.a.
Sldad1	LDDNLCGEIIRYGHFVEAAY	Missence	376 a.a.
#3-1	LDDNFTWRNNSIWTFC*	Frame shift	47 a.a.
#25-1	LDDNFTWRNNSIWTFC*<br>LDDNTRKNYSR*	Frame shift<br>Frame shift	47 a.a.<br>42 a.a.
#42-1	LDDNFTWRNNSIWTFC*<br>LDDNTRKNYSR*	Frame shift<br>Frame shift	47 a.a.<br>42 a.a.

C

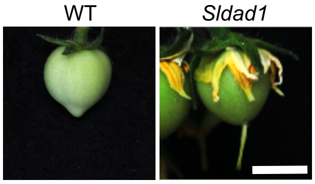

D

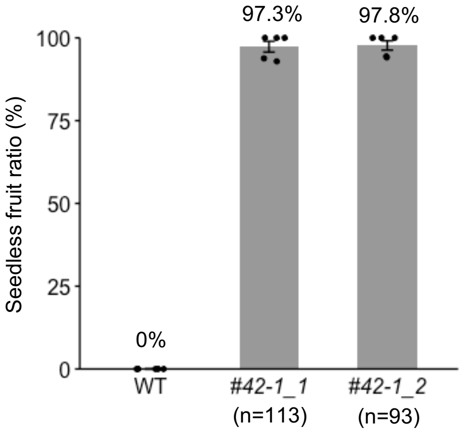

E

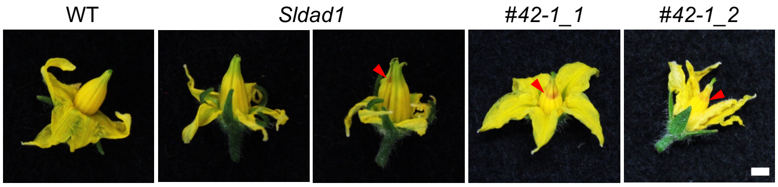

F

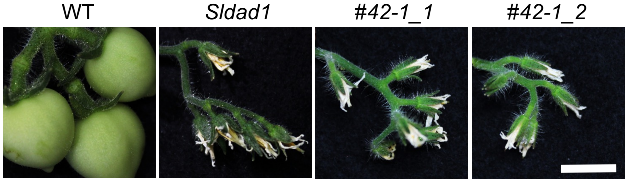

Fig. S8. *SlDAD1* mutations generated by CRISPR/Cas9. (A) Mutational patterns of sequences around gRNA1 and gRNA2 in T_0 transgenic lines. Blue indicates PAM sequences, and red indicates mutated sequences from the CRISPR/Cas9 system. (B) Amino acid sequence of T_0 transgenic lines. (C) Phenotype of fruits in WT and *Sldad1* mutant. In *Sldad1*, abscission of the anthers, petals, and styles did not occur. Scale bar is 1 cm. (D) Seedless fruit ratio of WT and *SlDAD1* knockout lines by CRISPR/Cas9 without emasculation. Two independent genome editing patterns were obtained in the T_2 generation. Data are mean (±SE), n=5. (E) Phenotype of flowers in WT, *Sldad1* and CRISPR/Cas9 mutants. Red arrowheads indicate browned anther cones. Scale bar is 2 mm. (F) Phenotype of inflorescences in WT, *Sldad1* and CRISPR/Cas9 mutants. The failure of ovary enlargement was observed in terminal inflorescences. Scale bar is 1 cm. DAA, days after anthesis.

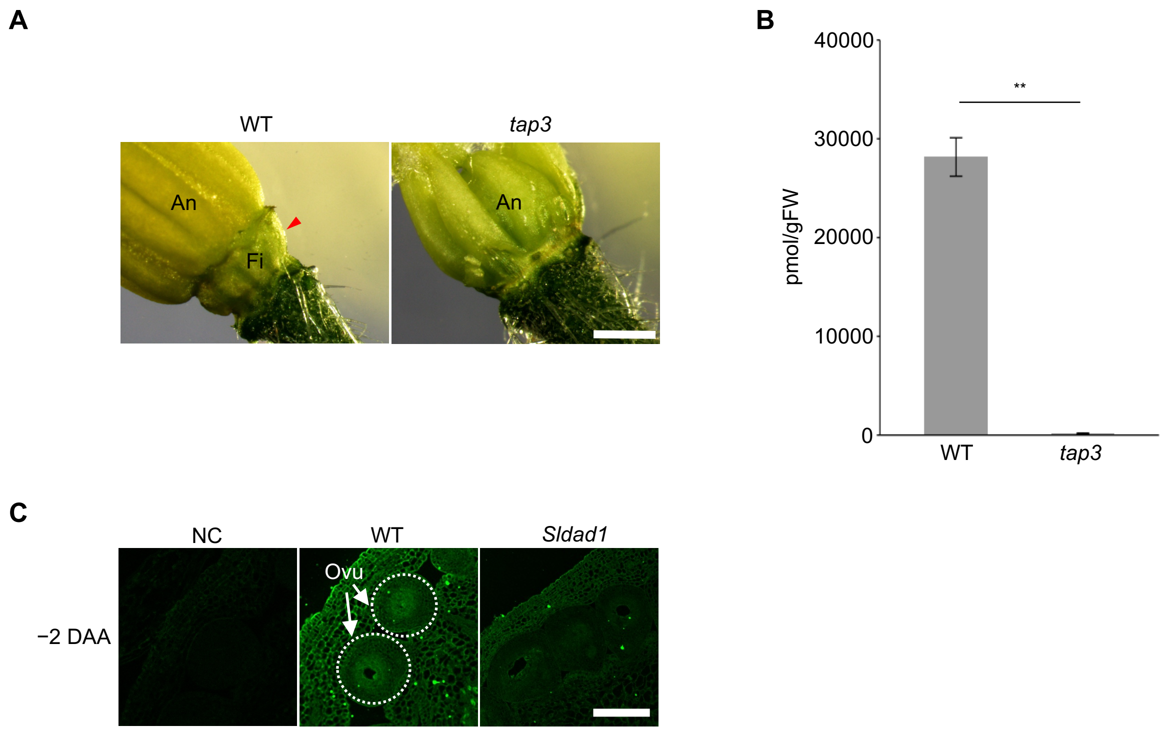

Fig. S9. Endogenous levels of JA in ovaries. (A, B) Phenotype and endogenous phytohormone level of JA in WT and *tomato apetala3* (*tap3*) mutant. (A) Stamen phenotype of WT and *tap3* mutant. WT formed both anther and stamen filament (red arrowhead), whereas *tap3* showed the lack of filament. Scale bar is 1 mm. (B) JA concentration in WT and *tap3* mutant ovaries at −2 DAA (Kusano *et al*., 2022; *Cells* 11, 1420). Data are mean (±SE), n=4. Asterisks indicate significant differences from WT at −2 DAA (**P<0.01; Welch t-test). (C) Replicates of immunocytochemical assay in ovaries of WT and *Sldad1* mutant. JA/JA-Ile was detected by an anti-JA antibody. As a negative control, the primary anti-JA antibody was omitted. Bar = 100 μm. An, anther; Fi, filament; Ovu, ovule; NC, negative control; JA, jasmonic acid; JA-Ile, JA-isoleucine; DAA, days after anthesis.

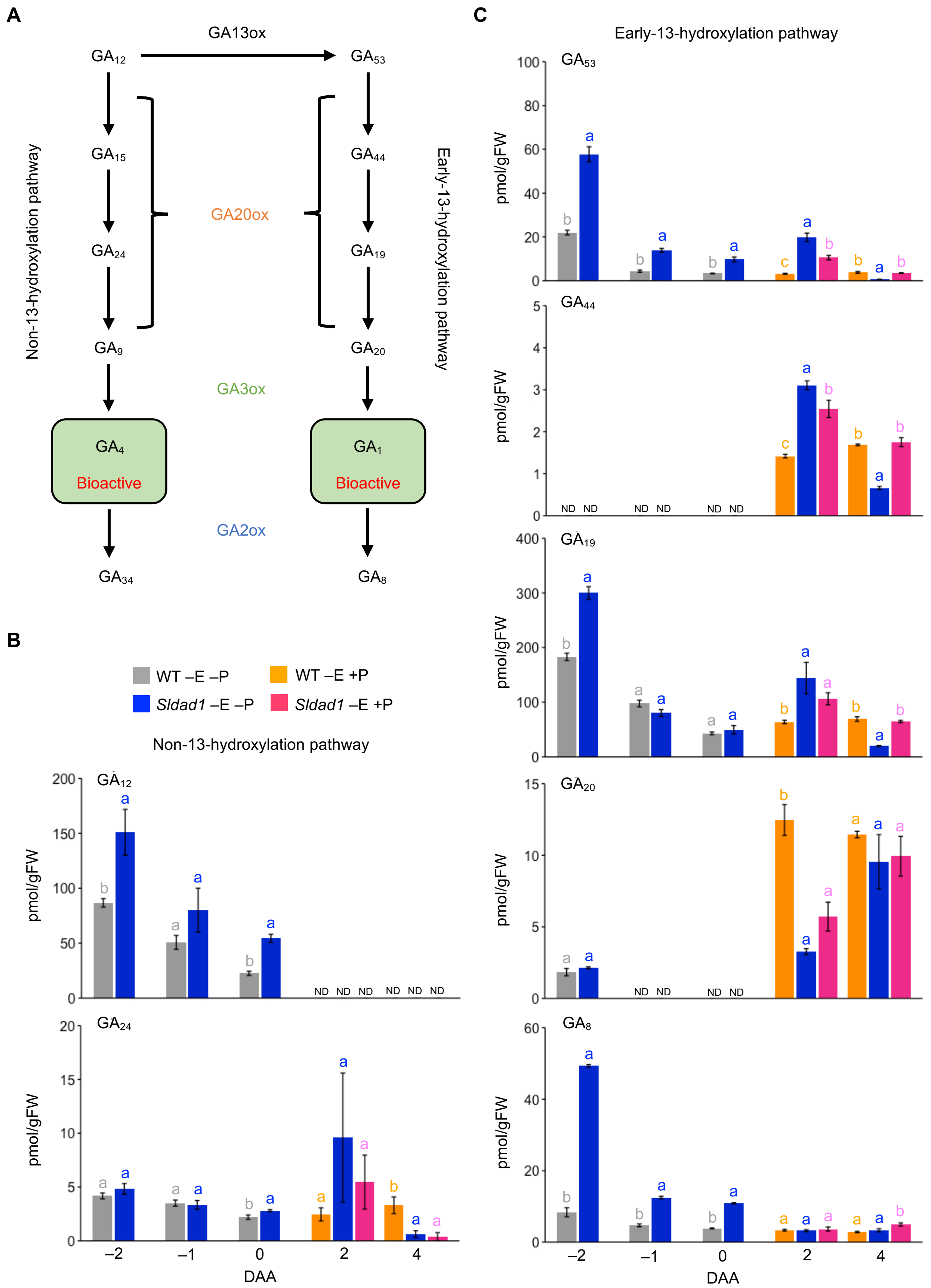

Fig. S10. Endogenous concentrations of GAs in WT and *Sldad1* ovaries. (A) Simplified GA biosynthesis pathway. (B, C) Concentrations of (B) GA$_4$ precursors (GA$_{12}$ and GA$_{24}$) and (C) GA$_1$ precursors (GA$_{53}$, GA$_{44}$, GA$_{19}$ and GA$_{20}$) and a catabolite (GA$_8$) concentration in the ovary. Data are mean (±SE), n=3–4. Different letters indicate significant differences at each time point (*P<0.05; Tukey–Kramer test). GA$_3$, GA$_7$, GA$_{15}$, GA$_9$ and GA$_{34}$ were not detected. GA, gibberellin; DAA, days after anthesis; –E, non-emasculated; +P, pollinated; ND, not detected.

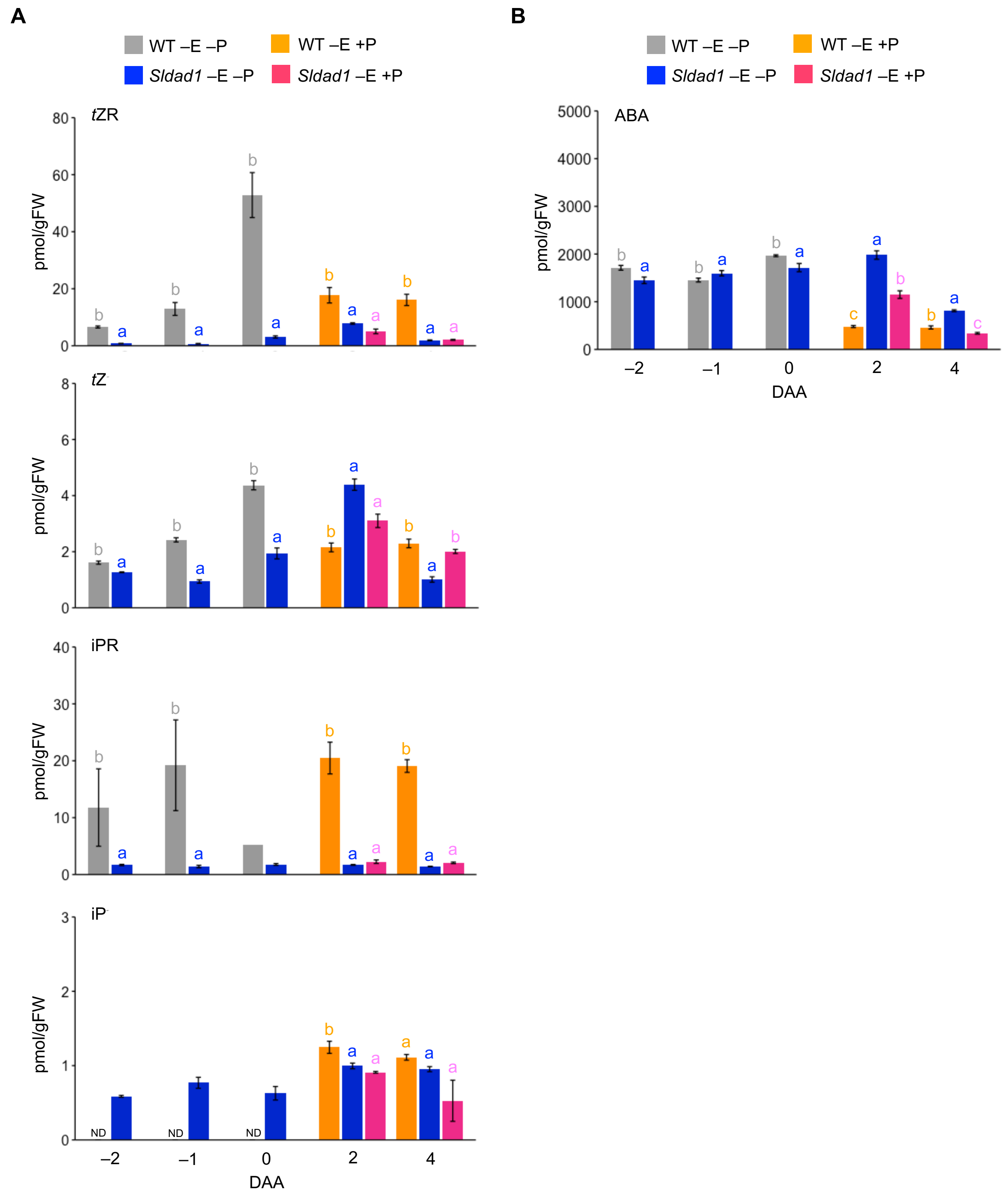

Fig. S11. Endogenous concentrations of CKs and ABA in WT and *Sldad1* ovaries. (A, B) Concentrations of (A) CKs (*t*ZR, *t*Z, iPR and iP) and (B) ABA concentration in the ovary. Data are mean (±SE), n=3–4. Different letters indicate significant differences at each time point (*P<0.05; Tukey–Kramer test). Values without error bars at some time points indicate the means between samples because the phytohormones were not detected in some samples. CKs, cytokinins; *t*ZR, *trans*-zeatin riboside; *t*Z, *trans*-zeatin; iPR, N^6- (Δ^2-isopentenyl) adeno-sine; iP, isopentenyladenine; ABA, abscisic acid; DAA, days after anthesis; –E, non-emasculated; +P, pollinated; ND, not detected.

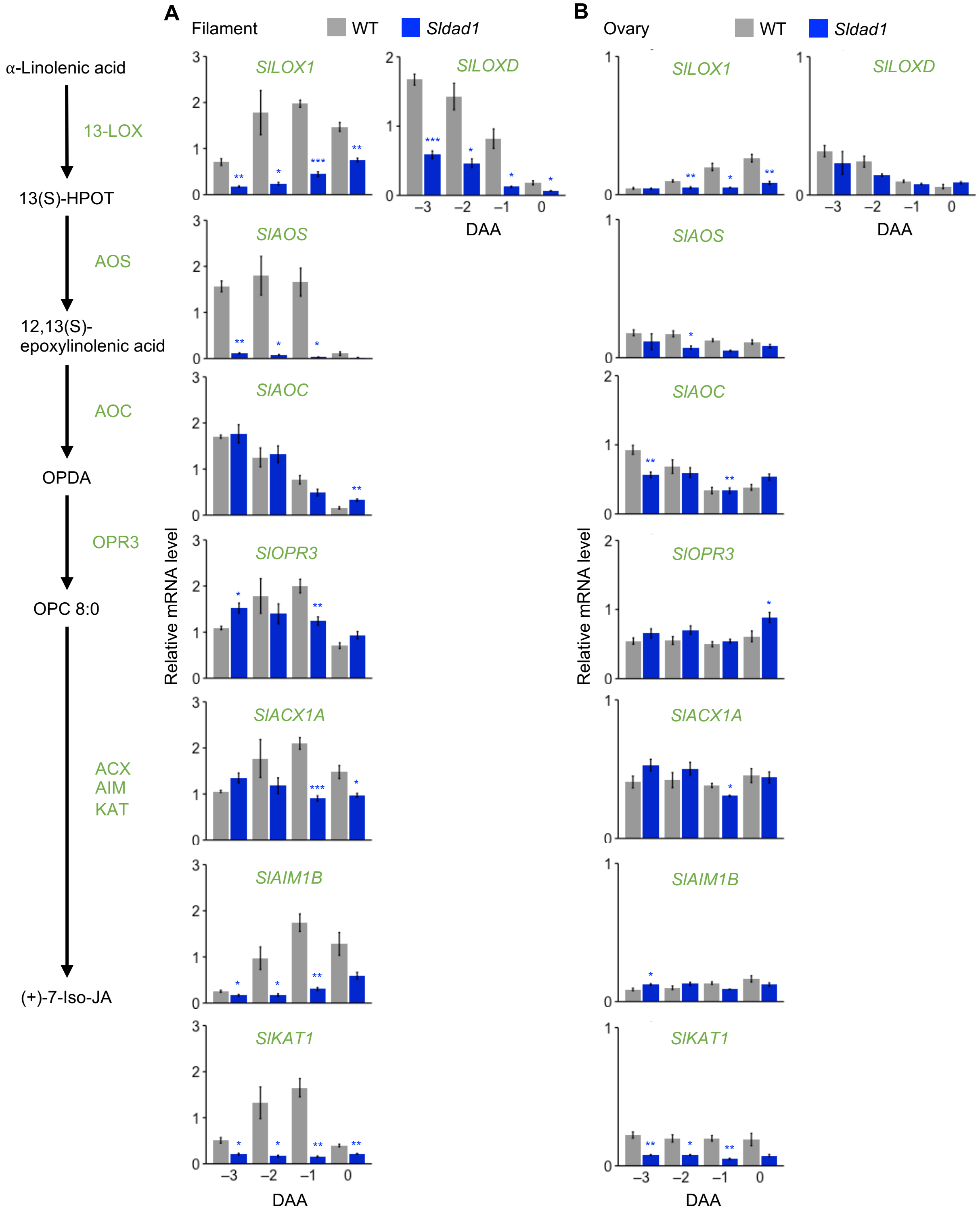

Fig. S12. Validation of expression of JA biosynthesis genes by qRT–PCR. (A, B) The mRNA levels of JA biosynthesis genes in filaments (A) and ovaries (B) of WT and *Sldad1* mutant from −3 to 0 DAA. The *SAND* gene was used as an internal control. Data are mean (±SE), *n*=4. Asterisks indicate significant differences (**P*<0.05, ***P*<0.01, ****P*<0.001; Welch *t*-test). LOX1, lipoxygenase 1; LOXD, lipoxygenase D; AOS, alleneoxidesynthase; AOC, alleneoxidecyclase; OPR 3, oxidoreductase 3; ACX, acyl-CoA oxidase; KAT, 3-keto-acyl-CoA thiolase; DAA, days after anthesis.

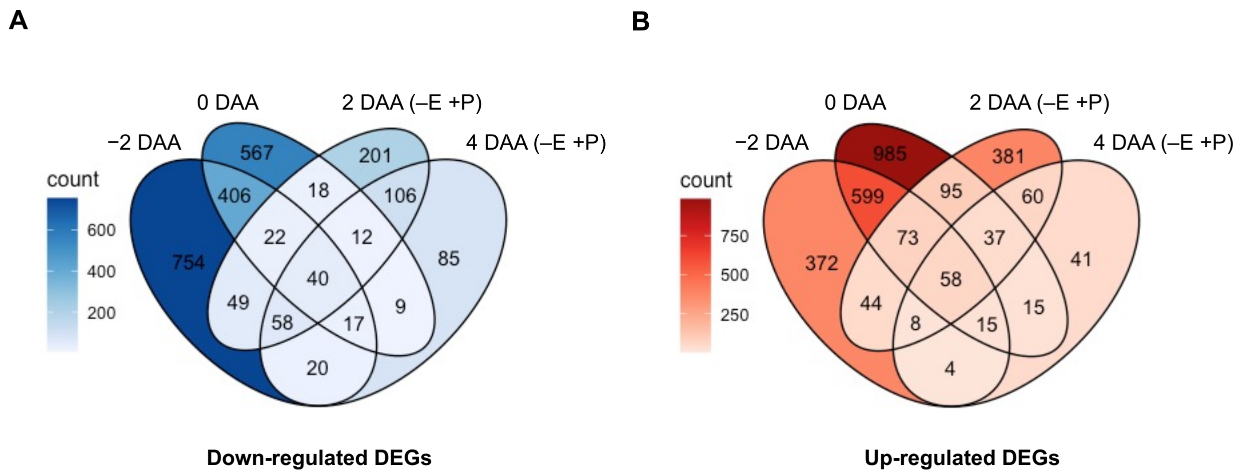

Fig. S13. Venn diagrams of up- and down-regulated DEGs in *Sldad1* ovaries at different time-points. (A) Numbers of up-regulated DEGs and (B) numbers of down-regulated DEGs. DAA, days after anthesis; –E, non-emasculated; +P, pollinated.

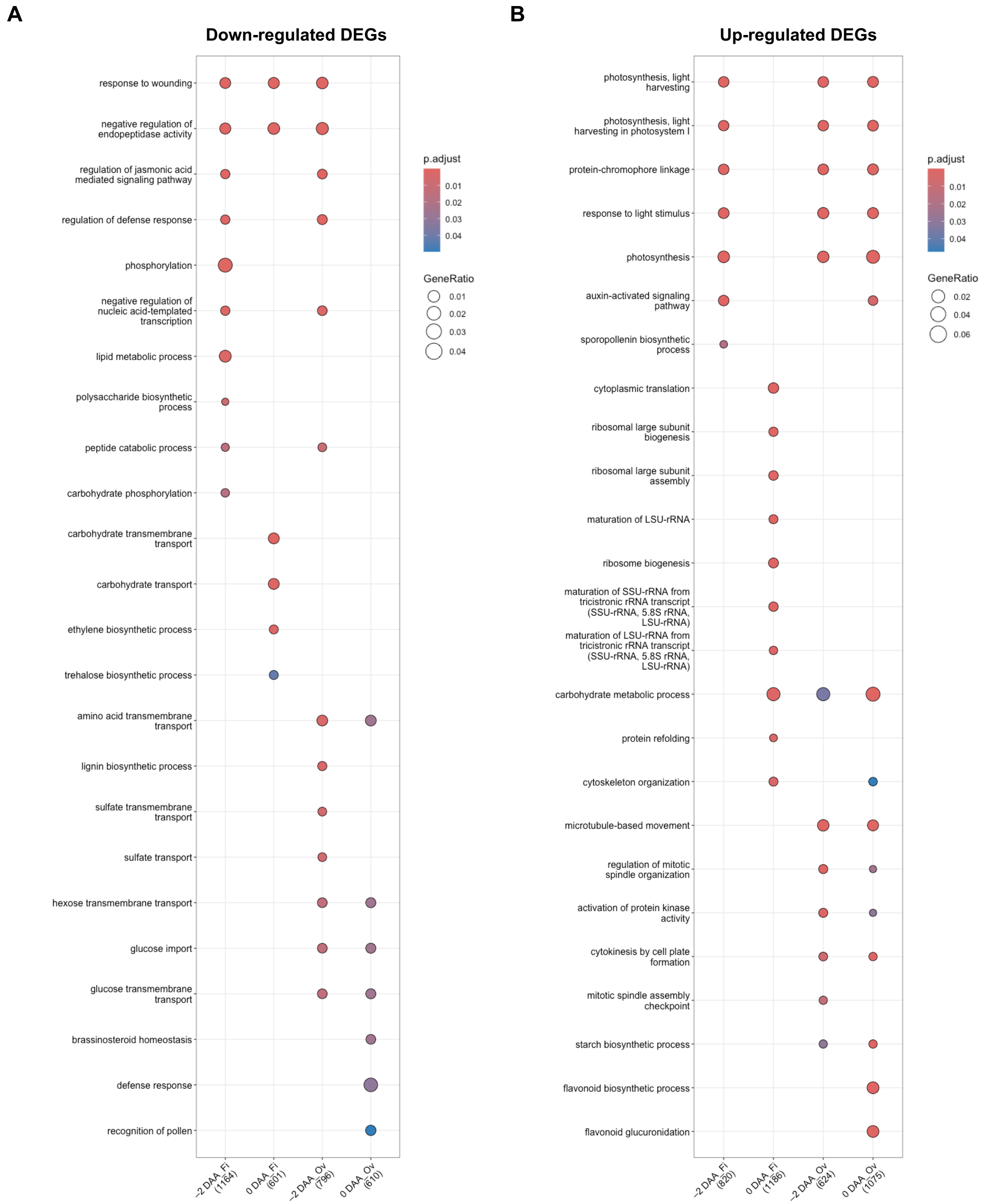

Fig. S14. GO enrichment analysis of non-emasculated WT and *Sldad1* ovaries from −2 DAA and 0 DAA. (A, B) Enriched GO terms of biological process in *Sldad1* mutant compared to WT. (A) Down-regulated and (B) up-regulated DEGs in *Sldad1* at each timepoint were used. A false discovery rate (FDR) of <0.05 was used as the criterion to obtain enriched GO terms. Numbers of DEGs enriched in GO terms are shown in parentheses. Fi, filament; Ov, ovary; DAA, days after anthesis.
